# 5‐HT_7_R Deficiency Alleviates ADP‐Heptose‐Induced Cognitive Impairment via Inhibiting Ferroptosis and Neuroinflammation in Mice

**DOI:** 10.1111/cns.70455

**Published:** 2025-06-12

**Authors:** Xiao Zou, Yu‐Xin Yang, Bing‐Jie Yue, Han‐Yinan Yang, Yan‐Rong Yang, Meng‐Yang Li, Ou Du, Gan Qiao, Yi‐Jin Wu, Jun‐Rong Du, Fang‐Yi Long

**Affiliations:** ^1^ Department of Pharmacology, Key Laboratory of Drug‐Targeting and Drug Delivery System of the Education Ministry and Sichuan Province, Sichuan Engineering Laboratory for Plant‐Sourced Drug and Sichuan Research Center for Drug Precision Industrial Technology, West China School of Pharmacy Sichuan University Chengdu Sichuan China; ^2^ Department of Epidemiology and Health Statistics, West China School of Public Health and West China Fourth Hospital Sichuan University Chengdu Sichuan China; ^3^ School of Pharmacy Southwest Medical University Luzhou China; ^4^ Laboratory Medicine Center Sichuan Provincial Women's and Children's Hospital, Affiliated Women's and Children's Hospital of Chengdu Medical College, Chengdu Medical College Chengdu China

**Keywords:** 5‐HT_7_R, ADP‐heptose, cognitive impairment, ferroptosis, microglia polarization, neuroinflammation

## Abstract

**Background:**

ADP‐heptose (ADP‐hep), a soluble intermediate in the biosynthesis of lipopolysaccharide in Gram‐negative bacteria, is known to trigger inflammation. Our research suggests that 5‐hydroxytryptamine receptor 7 (5‐HT_7_R) could serve as a potential pattern recognition receptor (PRR) for ADP‐hep, yet the precise mechanism of ADP‐hep's regulation on 5‐HT_7_R remains unclear.

**Aims:**

Based on the results of mRNA sequencing analysis, this study took ferroptosis of neurons and microglia as a starting point to explore the role and underlying mechanisms of ADP‐hep/5‐HT_7_R signaling in mediating neuroinflammation and cognitive impairment in mice.

**Results:**

We found that 5‐HT_7_R may act as a potential PRR for ADP‐hep and potentially bind to ADP‐hep. 5‐HT_7_R deficiency significantly ameliorated cognitive dysfunction induced by ADP‐hep in mice, as well as ferroptosis mediated by the p53/cystine‐glutamate antiporter (xCT)/glutathione peroxidase 4 (GPX4) signaling pathway and its associated key markers. Furthermore, 5‐HT_7_R deficiency inhibited ferroptosis in neurons and M2‐type microglia, mitigated the decline in the proportion of M2‐type microglia, and subsequently suppressed the inflammatory microenvironment to promote neuronal survival, thereby exerting neuroprotective effects.

**Conclusion:**

In summary, 5‐HT_7_R deficiency promotes cognitive recovery by alleviating the neuronal and microglial ferroptosis triggered by ADP‐hep, subsequently dampening the inflammatory microenvironment to support neuronal viability. These findings provide a novel perspective and approach for the development of innovative therapeutic strategies for the treatment of cognitive impairment‐related diseases.

## Graphical abstract

5‐HT_7_R deficiency can inhibit the abnormal changes of cAMP/PKA/MAPK signaling pathway and p53/xCT/GPX4 signaling pathway‐mediated ferroptosis caused by ADP‐hep in neuronal and M2 microglia, inhibit the inflammatory microenvironment, and promote neuronal survival, thereby improving cognitive dysfunction.

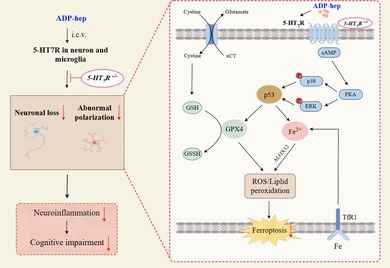

## 1 Introduction

Alzheimer's disease (AD), vascular dementia and traumatic brain injury, as common diseases of cognitive dysfunction, are characterized by high disability rate and high mortality. Their prevention and treatment and the improvement of patients' quality of life are still a major medical and social problems worldwide [1, 2]. Studies have confirmed that although the specific pathogenesis of cognitive dysfunction diseases is different, their pathogenesis is closely related to neuroinflammation. In particular, as the main immune effector cells of the central nervous system (CNS), the abnormal activation of microglia can release a large number of inflammatory factors, promote the development of inflammation in cerebral ischemic areas, and eventually lead to neuronal death and cognitive impairment [3, 4]. In addition, anti‐inflammatory treatments have been tried in clinical trials to prevent or slow down the progression of cognitive dysfunction disorders such as AD [5]. Therefore, in‐depth exploring neuroinflammation as a therapeutic target has broad prospects for improving cognitive impairment.

With the in‐depth study of its pathogenesis in recent years, it has been found that ferroptosis is closely related to the occurrence and development of neuroinflammation [6]. Ferroptosis is immunogenic and is accompanied by an inflammatory response. Ferroptosis is a kind of cell death caused by oxidative stress, and reactive oxygen species (ROS) accumulation is its typical characteristic. Excessive ROS can activate the nuclear factor‐kappa B (NF‐κB), NOD‐like receptor thermal proteindomain associated protein 3 (NLRP3) inflammasome, and TLR4 to trigger an inflammatory response [7, 8]. Damage associated molecular patterns (DAMPs) are endogenous molecules released after cellular damage or activation, also known as danger signals, including high mobility group protein B1 (HMGB1), heat shock proteins, and so on, and trigger immune responses by binding to pattern recognition receptors (PRRs). There is evidence that DAMPs released by ferroptosis can drive inflammatory cells to release tumor necrosis factor‐α (TNF‐α) and other inflammatory mediators in an NF‐κB‐dependent manner, causing an inflammatory response [9]. Research has demonstrated that the accumulation of iron within microglial cells can induce their activation, thereby exacerbating the progression of neuroinflammation. This process is associated with an increased generation of reactive oxygen species (ROS) and iron overload, culminating in neuronal ferroptosis and synaptic injury. Neuronal ferroptosis subsequently releases iron ions and damage‐associated molecular patterns (DAMPs), which further activate microglia, establishing a deleterious feedback loop that ultimately contributes to cognitive dysfunction [10, 11, 12]. In addition, elevated iron levels in brain areas also affect the phenotype and function of microglia. Studies have shown that in neurodegenerative diseases, microglia with different phenotypes show different sensitivity to ferroptosis, among which M2 microglia are highly sensitive to ferroptosis [13]. Based on the above research background, an in‐depth exploration of the molecular regulatory mechanisms of neuronal ferroptosis and M2‐type microglia ferroptosis is of great significance for the prevention and treatment of cognitive impairment‐related diseases.

Microglia are the first and most important line of immune defense in the CNS. They include the recognition of pathogen‐associated molecular patterns (PAMPs) by PRRs, the production of inflammatory cytokines, and the recruitment of immune cells to the site of infection [14]. So far, different families of PRRs have been identified, and their homologous PAMPs have been widely reported. For example, toll‐like receptors (TLRs) can sense a variety of microbial molecular signatures, including lipopolysaccharide (LPS), bacterial and viral DNA, and so on. C‐type‐lectin receptors (CLRs) detect carbohydrate structures, while RIG‐I‐like receptors (RLRs) detect the presence of viral RNA. Nucleotide‐binding oligomerization domain‐containing proteins 1 (NOD1) and NOD2 are two proteins in the NOD‐like receptor (NLR) family, identifying peptidoglycan motifs meso‐diaminopimelic acid or muramyldipeptide, respectively [15]. Recently, ADP‐heptose (ADP‐hep), as a soluble intermediate in the Gram‐negative bacteria LPS biosynthetic pathway, has been identified as a PAMP [16, 17, 18]. Moreover, ADP‐hep has been shown to regulate alpha protein kinase 1 (ALPK1, a novel cytoplasma PRR) and its downstream signaling pathways, including TIFA and TRAF6 proteins, and induce the activation of NF‐κB and the secretion of inflammatory cytokines [18]. Furthermore, we have shown in our previous studies that ADP‐hep can also activate 5‐hydroxytryptamine receptor 7 (5‐HT_7_R), a G‐protein‐coupled receptor (GPCR) mainly distributed in regions such as the hippocampus, prefrontal cortex, and thalamus, which can be coupled to adenylate cyclase by stimulating the GαS protein. This causes the second messenger cyclic adenosine monophosphate (cAMP) to accumulate within the cell and create a cascade reaction [19]. So, whether 5‐HT_7_R is also a PRR of ADP‐hep is unknown. The specific mechanism of ADP‐hep regulating 5‐HT_7_R and its role in cognitive impairment are still unclear.

It is well known that microglial activation is triggered during the neurodegenerative processes of cognitive impairment‐related diseases, and that overactivation of microglia produces large amounts of neuroimmune inflammatory factors, ultimately leading to neuronal damage and cognitive impairment [3, 4]. Therefore, on the basis of 5‐HT_7_R knockout mice, this study induced a mouse cognitive impairment model by ADP‐hep, and took neuronal ferroptosis and microglial ferroptosis as the starting point to explore the role and mechanism of ADP‐hep/5‐HT_7_R signaling‐mediated neuroinflammation in cognitive impairment in vivo and in *vitro*. The ADP‐hep/5‐HT_7_R signal may serve as a novel natural immune signaling pathway, providing a new strategy for the treatment of cognitive impairment‐related diseases, and laying a preliminary foundation for the subsequent development of corresponding targeted drugs in the future.

## 2 Materials and Methods

### 2.1 Chemicals and Reagents

ADP‐hep was purchased from J&K Scientific Ltd. (Beijing, China). LP‐211 and SB‐269 970 were purchased from MedChemExpress (New Jersey, USA). Dibutyryl‐cAMP (dbcAMP) and 3‐isobutyl‐1‐methylxanthine (IBMX) were purchased from Macklin (Shanghai, China). Pronase was obtained from Roche (Basel, Switzerland). Fetal bovine serum (FBS) and Dulbecco's modified Eagle's medium (DMEM)‐F1/2 were purchased from Gibco (CA, USA). CCK‐8 assay kit and sodium dodecyl sulfate polyacrylamide gel electrophoresis (SDS‐PAGE) rapid gel preparation kit were the products of Beyotime (Shanghai, China). Anti‐CD16/CD32, CD11b, and CD45 antibodies were bought from eBioscience (California, USA). Anti‐Iba‐1 antibody was purchased from Zhejiang Ousaisi Biotechnology Co. Ltd. (Hangzhou, Zhejiang, China). Anti‐5‐HT_7_R, neuronal nuclei (NeuN), synaptophysin (SYN), PKA, and CD206 antibodies were obtained from abcam (Cambridge, UK). Anti‐Histone H3, p53, transferrin receptor protein 1 (TfR1) and ferroportin 1 (FPN1) antibodies were supplied by Proteintech (Wuhan, Hubei, China). Anti‐arachidonic acid 12‐lipoxygenase (ALOX12), inducible nitric oxide synthase (iNOS), glutathione peroxidase 4 (GPX4) and cysteine/glutamate transporter (xCT) antibodies were supplied by Hua'an Biotechnology (Hangzhou, Zhejiang, China). Anti‐p38 and p‐p38 antibodies were obtained from Zen‐bioscience (Chengdu, Sichuan, China). Anti‐Ferritin antibody was purchased from ABclonal (Wuhan, Hubei, China). Mice enzyme‐linked immunosorbent assay (ELISA) kits for Arg‐1, interleukin‐10 (IL‐10) and cAMP assay kit were purchased from Guizhou Ruixin Biotechnology Co. Ltd. (Qianxinan buyi and miao autonomous prefecture, Guizhou, China). Mice ELISA kits for tumor necrosis factor‐α (TNF‐α) and IL‐6 tumor necrosis factor‐α (TNF‐α) were obtained from Dakewe Biotech Co. Ltd. (Shenzhen, Guangzhou, China). GSH, GSSG, superoxide dismutase (SOD), malondialdehyde (MDA), ROS, and mitochondrial membrane potential (JC‐1) assay kits were purchased from Beyotime Biotechnology (Shanghai, China). Cell ferrous ion assay kit was the product of Elabscience (Wuhan, Hubei, China). TRIzol reagent (Invitrogen, CA, USA).

### 2.2 Cells and Animals

The 5‐HT_7_R heterozygous mice were purchased from Cyagen Biosciences Inc. (Guangzhou, Guangdong, China). Male 5‐HT_7_R knockout (5‐HT_7_R^−/−^) mice (6–8 weeks, 20–25 g) and littermate wild‐type (WT) mice (6–8 weeks, 20–25 g) were selected by mouse genotype identification for subsequent experiments. Mice were maintained on a 12‐h light/dark cycle under specific pathogen‐free conditions. The animal experiment was approved by the Animal Experimentation Ethics Committee of Sichuan University (K2024026). All care and treatment of experimental animals were in strict accordance with the guidelines of the experimental animal management requirements of Sichuan University. Primary microglia and neuron cells were isolated from the brain of newborn mice.

### 2.3 Lateral Ventricle Stereotactic Injection

Mice were anesthetized with isoflurane through a gas anesthesia machine, and the mice were fixed in a prone position on a brain stereotaxator. According to the map of mouse brain location, an implantable cannula (RWD Life Science) was inserted into the left cerebral ventricle. The cannula was placed using these stereotaxic coordinates: 1.0 mm lateral to the midline, 2.5 mm ventral from the skull surface, and 0.23 mm posterior to the Bregma. To keep the cannula free from blockage and securely in place, a guide cannula with a dummy cannula (a placeholder that prevents occlusion when not in use) was utilized. The assembly was then permanently affixed to the skull with dental cement, providing a stable platform for subsequent brain injections. The injections included either ADP‐hep at 0.5 mg/kg/day or vehicle (PBS) for 7 days. The body temperature of the mice was maintained using a small animal temperature maintainer throughout the operation. During the whole animal experiment period, the Y‐maze experiment was carried out on mice on the 8th day. The Passive avoidance experiment was conducted on days 9 to 10, and the Morris water maze (MWM) experiment was conducted on days 11 to 16. Finally, the mice were killed on the 17th day after anesthesia, and the brain tissue was taken for subsequent experiments. It is worth noting that brain tissues of mice were rapidly collected immediately after decapitation and flash‐frozen in liquid nitrogen to minimize the impact on various proteins and biochemical indicators (e.g., cAMP).

### 2.4 Preparation of Single‐Cell Suspension of Brain Tissue and Sorting of Microglia

The mice were anesthetized with isoflurane, then underwent cardiac perfusion with 0.01 M precooled PBS solution after thoracotomy. Then, in a sterile environment, the removed brains were cleaned, quickly cut, and transferred to a centrifugal tube containing digestive enzymes, and incubated on a constant temperature shaker for 60 min. The brain tissue solution was filtered with a 70 μm cell strainer, centrifuged at 300 *g* for 5 min, and the upper clarified liquid was taken. After mixing with percoll solution, the cells were left to precipitate after centrifugation at 950 *g* for 20 min. The cells were washed with Hank's balanced salt solution (HBSS) for 1–2 times, and finally the cells were precipitated with 100 μL HBSS solution, which was the single cell suspension of brain tissue.

The above cells were subjected to flow cytometry sorting. In this experiment, the cells were first blocked with CD16/CD32 (1:100) antibody. They were then dyed with PerCP‐Cyanine5.5‐CD11b (1:500) and PE‐CD45 (1:500) antibody and fed to a flow cytometer. CD11b^+^CD45^+^ cells were identified as microglia.

### 2.5 mRNA Sequencing Analysis

Total RNA was extracted using the TRIzol reagent according to the manufacturer's protocol. Then the libraries were constructed using VAHTS Universal V6 RNA‐seq Library Prep Kit according to the manufacturer's instructions. The transcriptome sequencing and analysis were conducted by OE Biotech Co. Ltd. (Shanghai, China). The libraries were sequenced on anllumina Novaseq 6000 platform and 150 bp paired‐end reads were generated. The clean reads were mapped to the reference genome using HISAT2. Fragments per kilobase of exon model per million mapped fragments (FPKM) of each gene was calculated and the read counts of each gene were obtained by HTSeq‐count. Differential expression analysis was performed using the DESeq2. Q value < 0.05 and fold change > 2 or fold change < 0.5 was set as the threshold for significantly differential expression gene (DEGs). Hierarchical cluster analysis of DEGs was performed using R (v 3.2.0) to demonstrate the expression pattern of genes in different groups and samples. Based on the hypergeometric distribution, GO, KEGG pathway, Reactome and WikiPathways enrichment analysis of DEGs were performed to screen the significant enriched term using R (v 3.2.0), respectively. Gene Set Enrichment Analysis (GSEA) was performed using GSEA software.

### 2.6 Molecular Docking

In general, the crystal structure of protein was downloaded from RCSB (https://www.rcsb.org/). However, since there is no complete 5‐HT_7_R 3D result in the database, we used the AlphaFold 2 python tool to build its 3D structure and finally determined the 3D model structure of 5‐HT_7_R based on the predicted local distance difference test (pLDDT) model evaluation. On the other hand, the mol file of ADP‐hep was prepared from ChemSpider 3D (ChemSpiderID: 26331769). The ADP‐hep structure was converted into a standard delay format (SDF) file and uploaded as a ligand. The PDB file of 5‐HT_7_R was uploaded as a target protein directly. These files were then checked and converted to pdbqt files by OpenBabel and MGLTools. CBDock analyzed all cavities of the protein. Afterwards, the molecular simulation docking procedure was initiated by using all the default parameters. After the docking, the binding sites of ligands and proteins were labeled by Chimera, and the binding analysis tool was used for evaluation and analysis. The BIOVIA Discovery Studio visualizer was used to analyze and label specific parameters of bonding interactions involved in binding affinity between ligands and receptors.

### 2.7 Drug Affinity Responsive Target Stability (DARTS) Assay

The DARTS assay was conducted according to the previously described protocol [20]. In short, the collected microglia cells were lysed with M‐PER buffer containing protease inhibitor and centrifuged at 12,000 rpm for 20 min. The supernatant was retained, and the TNC buffer was added. Lysates were equally divided into three parts for 2 h at room temperature with PBS or ADP‐hep (100 μM) and added with pronase (pronase:protein = 1:100) for 30 min. The reaction was terminated by adding EDTA and analyzed via Western blot.

### 2.8 Cellular Thermal Shift Assay (CETSA)

The CETSA assay was conducted according to the previously described protocol [21]. Briefly, the collected microglia cells were lysed with kinase buffer, centrifuged at 16100g for 20 min, and the supernatant was retained. Then, microglia cell lysates were divided into two groups, PBS group and ADP‐hep (100 μM) group. Each group was divided into 8 equal parts and heat treated at 35°C, 40°C, 45°C, 50°C, 55°C, 60°C, 65°C, and 70°C for 3 min, respectively. Then, samples were centrifuged for 10 min at 12,000 rpm in 4°C, the supernatants were collected, the protein loading buffer was added, and analyzed via Western blot.

### 2.9 Y‐Maze Experiment

Y‐maze experiment is a behavioral test for assessing short‐term working memory in animals. The Y‐maze is a horizontal maze consisting of three arms with an angle of 120° to each other. The three arms of the Y‐maze were numbered A, B, and C, and the mice were placed at the starting point of A, and the order of entering each arm within 5 min was recorded. The number of alternations was recorded: the sequence in which the mice entered three different test arms (BCA, CAB or ABC) in succession; other sequences were recorded as invalid. The calculation formula is alternation (%) = (number of alternations/maximum number of alternations) × 100% [22].

### 2.10 Passive Avoidance Experiment

Passive avoidance experiment is a behavioral test to evaluate short‐term working memory in animals. Firstly, the jumping platform test box equipped with a circular insulated rubber platform was preheated for 2 min. The mice were placed on an insulated rubber platform and timed for 5 min. When mice spontaneously explore external space, they will jump off the insulated rubber platform. After feeling the electrical stimulation, due to the initial contact with the copper column, they have not established a solid learning memory to avoid harm, so the mice sustained electric shock for 3 s or more; it is necessary to put the mice back on the insulated rubber platform to avoid long‐term electric shock to the mice. After 24 h, the test indicators were recorded: incubation period (the time when the mice first jumped off the platform) and the number of errors (the total number of jumps off the platform within 5 min) [23].

### 2.11 MWM Experiment

The experiment was divided into two parts. The first part is the hidden platform experiment: the experiment period is 5 days. The mice were trained and tested in a fixed quadrantal sequence at a fixed time of day. The measured time was 60 s, during which the mice were considered to have found the platform if they stayed on the safe platform for more than 5 s, and if the safe platform was not found within 60 s, the mice were guided to stay on the platform for 30 s. The escape latency period was the time it took the mice to find the platform, or 60 s if no platform was found. The second part is the space exploration experiment: the experiment was conducted 24 h after the end of the hidden platform experiment, the safe platform was withdrawn, and the mice entered the water from the opposite quadrant of the quadrant where the safe platform was located. The EthoVision XT trajectory analysis system was used to monitor mouse motion trajectories. The measurement time was 60 s, and the crossing times of the mice and the effective quadrant rate were recorded, so as to evaluate the spatial memory ability of the mice [22].

### 2.12 Culture of Primary Neuronal Cells

The newborn mice were sterilized by soaking in a 75% alcohol solution. On a sterile operating table, the mouse brain was removed and cleaned with a pre‐cooled HBSS solution. The tissue is then enzymatically dissociated using trypsin, supplemented with DNase to minimize cell clumping, and mechanically triturated to achieve a homogeneous cell suspension. This suspension is filtered through a cell strainer to remove debris. Cells are plated on dishes coated with poly‐D‐lysine to facilitate adhesion and cultured in neurobasal medium with 5% serum, B27, and N2 supplements in a humidified incubator at 37°C and 5% CO_2_.

The primary neuronal cells of wild type (WT) and 5‐HT_7_R knockout (5‐HT_7_R^−/−^) mice were extracted and inoculated into 24‐well plates. Then the cells were stimulated with 0.01 M PBS (vehicle) and ADP‐hep (100 μM) respectively for 24 h in order to facilitate subsequent experiments.

### 2.13 Culture of Primary Microglial Cells

The newborn mice were sterilized by soaking in a 75% alcohol solution. On a sterile operating table, the mouse brain was removed and cleaned with a pre‐cooled HBSS solution. Then, the meninges and blood vessels of the mouse brain were removed under the asana microscope and transferred to the EP tube containing pancreatic enzyme to rapidly cut up the brain tissue. The solution containing brain tissue fragments was digested in an incubator at 37°C for 10 min. The brain tissue solution was then filtered with a 100 μm cell strainer, and the filtrate was centrifuged at 303g for 8 min. The cell suspension was then filtered with a 40 μm cell strainer and centrifuged at 167g for 5 min to obtain cell precipitation. The cell precipitation was resuspended with DMEM‐F12 complete culture medium containing 10% FBS and inoculated into cell culture bottles, placed in cell incubators, and periodically replaced with fresh complete culture medium. At 10–12 days after inoculation, a large number of astrocyte adherent growths were observed under the microscope, and round microglia grew on top of astrocytes with loose attachment. Then the mixed glial cells were separated by the upright hand method, and the collected cell suspension was centrifuged at 1000 rpm for 8 min, and the resulting cell precipitate was primary microglia cells.

The primary microglia cells of wild type (WT) and 5‐HT_7_R knockout (5‐HT_7_R^−/−^) mice were extracted and inoculated into 24‐well plates. Then the cells were stimulated with 0.01 M PBS (vehicle) and ADP‐hep (100 μM) respectively for 24 h in order to facilitate subsequent experiments, and their conditioned medium (CM) was collected.

### 2.14 Culture of BV2 Cells

The BV2 cell line, derived from immortalized murine microglial cells, was purchased from the Institute of Basic Medical Sciences, Chinese Academy of Medical Sciences (Beijing, China). The cells were cultured in Dulbecco's Modified Eagle Medium (DMEM) supplemented with 10% fetal bovine serum (FBS) and 1% penicillin–streptomycin, maintained in a humidified incubator at 37°C with 5% CO2. (1) For the temporal dynamics experiment: BV2 cells were divided into four groups and incubated with ADP‐hep (100 μM) for 0 h, 6 h, 12 h, and 24 h, respectively. (2) For the effects of 5‐HT_7_R agonist, dbcAMP, and 5‐HT_7_R antagonist on ADP‐hep induced ferroptosis, BV2 cells were divided into seven groups: Control, ADP‐hep (100 μM), LP‐211 (10 μM), ADP‐hep (100 μM) + LP‐211 (10 μM), dbcAMP (30 μM), ADP‐hep (100 μM) + dbcAMP (30 μM), SB‐269970 (10 μM); following 24 h of grouped treatment, the cells were subjected to subsequent experiments.

### 2.15 The Effect of CM From Primary Microglia on Primary Neuronal Survival

Primary neuronal cells derived from WT mice were inoculated into 96‐well plates and incubated at 37°C for 24 h. At the end of the incubation, the old medium was replaced with primary microglial cells CM, and the incubation was continued for 24 h. Then 10 μL of CCK‐8 solution was added to each well, and the absorbance value was detected at 450 nm after continuing incubation for 2 h for calculating the survival rate of primary neuronal cells.

### 2.16 Immunofluorescence, ELISA and Biochemical Kit Assays

Brain sections and primary microglia cells of WT and 5‐HT_7_R^−/−^ mice were fixed in 4% formaldehyde solution for 30 min and soaked in 0.3% Triton X‐100 solution for 30 min for membrane permeabilization, then incubated with antigen repair solution for 10 min. After being treated with antigen repair and bovine serum albumin solutions, they were then incubated with appropriate concentrations of anti‐Iba‐1 (1:500), 5‐HT_7_R (1:700), NeuN (1:100), ferritin (1:100), FTH (1:100), p53 (1:500), GPX4 (1:100), iNOS (1:100) or CD206 (1:100) antibodies, goat anti‐mouse IgG (H + L) secondary antibody (1:300), goat anti‐rabbit IgG (H + L) cross‐adsorbed secondary antibody (1:500) or rabbit anti‐guinea pig IgG (H&L) secondary antibody (1:500), and DAPI in sequence. Images were collected using a fluorescence microscope. As for the detection of mitochondrial membrane potential, it was tested according to the instructions: 1 mL JC‐1 staining solution was added to the cell slide and incubated in a cell incubator at 37°C for 20 min. After washing with JC‐1 dyeing buffer, an appropriate amount of anti‐fluorescence sealer was added to the sealer, and the pictures were collected under a fluorescence microscope.

The levels of cAMP, SOD, GSH/GSSG, MDA, and Fe^2+^ in brain tissue, cAMP, TNF‐α, IL‐6, Arg‐1, and IL‐10 in cell culture supernatant, and intracellular MDA, SOD, GSH/GSSG, and Fe^2+^ in each group were detected according to the instructions of the respective ELISA/biochemical kit. During cAMP level measurement, to prevent the rapid degradation of cAMP during sample preparation, we added IBMX to maintain the stability of cAMP.

### 2.17 CCK‐8 Assay

Cell viability was assessed using the Cell Counting Kit‐8 (CCK‐8, Dojindo, Japan) according to the manufacturer's instructions. Briefly, after treatment, 10 μL of CCK‐8 solution was added to each well, and the cells were incubated for an additional 2 h at 37°C in a humidified atmosphere containing 5% CO_2_. The absorbance was measured at 450 nm using a microplate reader (Thermo Fisher Scientific, USA). The relative cell viability was calculated as a percentage of the control group, which was set to 100%.

### 2.18 Transmission Electron Microscopy (TEM)

The primary microglia cells were extracted and cultured according to the above method, and then fixed with 2% glutaraldehyde. Ultrathin sections (50 nm) were prepared, stained with 3% aqueous uranyl acetate for 1 h, and counterstained with 0.3% lead citrate. The sections were analyzed by TEM.

### 2.19 Quantitative Reverse Transcription‐Polymerase Chain Reaction (RT–qPCR)

The above cell samples were lysed in TRNzol reagent. Total RNA was extracted by chloroform, purified by isopropanol and 75% ethanol. We further used the RevertAid RT reverse transcription kit to reverse transcribe RNA to obtain cDNA. Finally, quantitative PCR was performed on the Roche LightCycler LC480 Real‐Time PCR system (Basel, Switzerland). Using GAPDH as the internal reference, the relative mRNA expression was calculated by 2^−ΔΔCT^ [24, 25]. The sequences of the gene‐specific primers (from 5′ end to 3′ end) used were as follows: Htr7‐forward, 5′‐TGCGGGGAGCAGATCAACTA‐3′, and reverse, 5′‐GACAAAGCACACCGAGATCAC‐3′; Ptgs2‐forward, 5′‐TGAG CAACTATTCCAAACCAGC‐3′, and reverse, 5′‐GCACGTA GTCTTCGATCACTATC‐3′; Irak3‐forward, 5′‐CTGGCTGGATG TTCGTCATATT‐3′, and reverse, 5′‐GGAGAACCTCTAAAA GGTCGC‐3′; GAPDH‐forward, 5′‐AGCAGTCCCGTACACTGGC AAAC‐3′, and reverse, 5′‐TCTGTGGTGATGTAAATGTCCTCT‐3′.

### 2.20 Western Blot (WB) Assay

The total proteins of cells and tissues were extracted with a pre‐prepared solution of protein‐cracking complex. The nucleoprotein of the tissue was extracted by a subcellular structure nucleoplasmic protein extraction kit. The samples were quantified with a BCA kit and then SDS‐PAGE was performed on a standardized protein sample (10 μg per lane) [26, 27]. The isolated proteins were transferred to polyvinylidene fluoride (PVDF) membrane and sealed with 5% skim milk at room temperature for 1 h. The PVDF membrane was incubated with different concentrations of primary antibodies (anti‐5‐HT_7_R antibody, 1:1000; anti‐p38 antibody, 1:2000; anti‐p‐p38 antibody, 1:2000; anti‐SYN antibody, 1:2000; anti‐PKACβ antibody, 1:5000; anti‐p53 antibody, 1:1000; anti‐ALOX12 antibody, 1:1000; anti‐CD206 antibody, 1:1000; anti‐iNOS antibody, 1:1000; anti‐GPX4 antibody, 1:5000; anti‐TfR1 antibody, 1:5000；anti‐FPN1 antibody, 1:1000; anti‐xCT antibody, 1:5000; anti‐H3 antibody, 1:5000; anti‐β‐actin antibody, 1:1000) at 4°C overnight. To ensure accurate quantification, β‐actin was used as a loading control. Then the PVDF membrane and the corresponding secondary antibodies (anti‐rat or rabbit HRP‐linked IgG antibody, 1:10000) were incubated in a constant temperature shaker at 37°C for 1 h. Finally, we used a gel imaging system to photograph the PVDF membrane under enhanced chemiluminescence (ECL) solution and analyzed the gray values of its bands using ImageJ (version 1.52a, NIH, Bethesda, MD, USA). Due to the large number of target proteins in this study, we excised target proteins with significantly different molecular weights from the same membrane and incubated them with their respective specific antibodies separately. Therefore, these target proteins (including ALOX12, xCT, GPX4, TfR1, and FPN1) share the same β‐actin as a loading control. For some target proteins (including 5‐HT7R, PKACβ, p38, and p‐p38) that have molecular weights too close to β‐actin to allow membrane excision, we stripped the membrane with protein stripping buffer before re‐probing with new target antibodies. As for nuclear proteins (p53), we used Histone H3 as the internal reference.

### 2.21 Statistical Analysis

All data were presented as mean ± standard error mean (SEM). Data analysis was performed using SPSS 26.0 (Chicago, IL, USA) software. When grouped into two groups, independent sample *t*‐tests are used. When the grouping is greater than or equal to three groups, one‐way analysis of variance (ANOVA) was used; *LSD* test was used for homogeneous variance, and *Tamhani T2* test was used for uneven variance. Two‐way ANOVA was used when there are multiple variables, and Bonferroni–Holm was used to conduct the post hoc test. Differences were considered statistically significant when *p* < 0.05.

## 3 Results

### 3.1 5‐HT_7_R Is a Potential Target for ADP‐Hep

**FIGURE 1:**
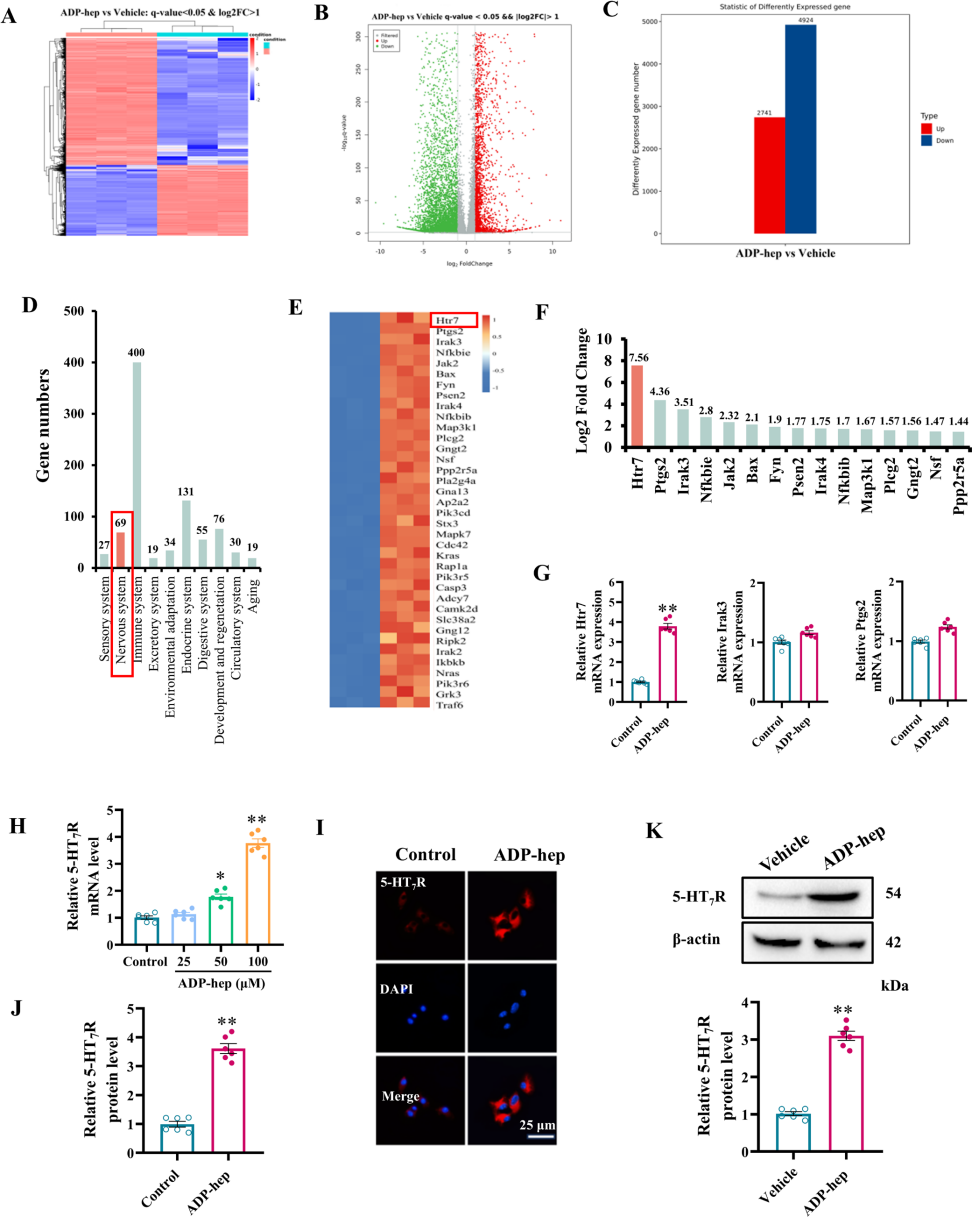
5‐HT_7_R Is a potential target of ADP‐hep by mRNA sequencing analysis. (A) Cluster heatmap of differentially expression gene. (B) Volcano plot of differentially expression gene. (C) Column chart of differentially expression gene. (D) KEGG‐organic systems analysis column chart of up‐regulated DEGs. (E) Heatmaps of up‐regulated differentially expressed genes in nervous system. (F) Sequence diagram of log_2_ fold change in nervous system (top 15). (G) The mRNA levels of related gene in mouse brain microglia. (H) The mRNA levels of 5‐HT_7_R in primary microglia cells treated with ADP‐hep (0, 25, 50, and 100 μM) for 24 h. (I, J) The relative expression of 5‐HT_7_R in primary microglia cells were detected by immunofluorescence after ADP hep (100 μM) treatment for 24 h. (K) The expression of 5‐HT_7_R in brain tissue in different groups of mice, the ratio of 5‐HT_7_R to β‐actin were analyzed. The data were expressed as mean ± SEM. (*n* = 6/group for H–K). Data were analyzed with two‐tailed Student's *t*‐test (G, J). Data were analyzed with one‐way ANOVA followed by *LSD* or *Tamhani T2* post hoc test (H). *p < 0.05, **p < 0.01 vs. vehicle or control.

In the previous experiment, different concentration doses of ADP‐hep (0.25, 0.5, 1 and 2 mg/kg) were injected into the lateral ventricle of mice (Figure S1A), and the levels of TNF‐α and IL‐6 in the brain of mice were detected (Figure S1B) to evaluate the effects of different concentrations of ADP‐hep in a dose‐dependent manner on neuroinflammation in mice (Figure S1B). Finally, a dose of 1 mg/kg was selected for the follow‐up experiment. Microglia, as the most important immune effector cells in the central nervous system (CNS), mediate neuroinflammation and play a very important role in the injury and disease outcome of the CNS [28]. In order to further explore the effects of ADP‐hep on neuroinflammation in mice, microglia were sorted by flow cytometry (Figure S2), and the effects of ADP‐hep on mRNA expression profiles of mouse brain microglia were explored by mRNA sequencing analysis. Microglia at different induce‐statuses showed the distinctively different gene expression profiles. The differentially expressed genes (DEGs) between ADP‐hep induced microglia and vehicle induced microglia were exhibited in Figure 1A. There were 2741 up‐regulated genes and 4924 down‐regulated genes in DEGs (Figure 1B,C). Here, we focus on the up‐regulated DEGs in the nervous system (Figure 1D,E), and plotted the top 15 genes with Log2 FC values. The first ranked gene was *Htr7* (Figure 1F), suggesting that ADP‐hep may play a series of roles by regulating *Htr7*. However, the specific mechanism by which ADP‐hep regulates *Htr7* is unknown. Therefore, based on the results of mRNA sequencing analysis, we further analyzed the effect of ADP‐hep on *Htr7* signaling, and the results of KEGG‐human diseases showed that up‐regulated DEGs were significantly enriched in neurodegenerative diseases and AD (Figure S3A). It is suggested that ADP‐hep may affect CNS diseases. KEGG‐environmental information processing analysis showed that up‐regulated DEGs were significantly enriched in MAPK signal pathway, TNF signal pathway, NF‐κB signal pathway, cAMP signal pathway, and neuroactive receptor ligand interaction (Figure S3B), suggesting that the above enriched signal pathways may be downstream of *Htr7*. The KEGG‐cellular processes results showed that up‐regulated DEGs were enriched in cellular processes such as p53 signal pathway and ferroptosis (Figure S3C), suggesting that ADP‐hep may regulate p53 signaling and thus affect ferroptosis. At the same time, we conducted GO enrichment analysis on the up‐regulated DEGs, and the results showed that DEGs were significantly enriched in inflammatory immune response, tumor necrosis factor‐activated receptor activity, G‐protein‐coupled receptors, and other signaling pathways (Figure S3D and E). Moreover, the results of gene set enrichment analysis showed that up‐regulated DEGs were significantly enriched in inflammatory response, TNF signal, NF‐κB signal, and other gene sets (Figure S3F–H). Based on the results of the above mRNA sequencing analysis, we speculate that ADP‐hep may affect cAMP release by acting on 5‐HT_7_R (gene name: *Htr7*), then regulate the MAPK signaling pathway, and further interfere with the p53‐mediated ferroptosis, thereby exerting an inflammatory response.

To further validate the above conclusion, we first detected the mRNA levels of *Htr7*, *Ptgs2*, and *Irak3* in the sorted microglia cells. The results showed that compared with the vehicle group, ADP‐hep significantly up‐regulated the mRNA levels of *Htr7* in sorted microglia cells (Figure 1G). Moreover, ADP‐hep dose‐dependently increased the mRNA level of *5‐HT*_*7*_ *R* (Figure 1H). The immunofluorescence results of mice primary microglia showed that ADP‐hep promoted the protein expression of 5‐HT_7_R (Figure 1I,J), and WB results in vivo further verified the above results (Figure 1K). The above results suggest that 5‐HT_7_R may also be a potential receptor for ADP‐hep; that is, ADP‐hep can activate a series of downstream inflammatory reactions through 5‐HT_7_R.

### 3.2 ADP‐Hep May Bind Directly to 5‐HT_7_R

**FIGURE 2:**
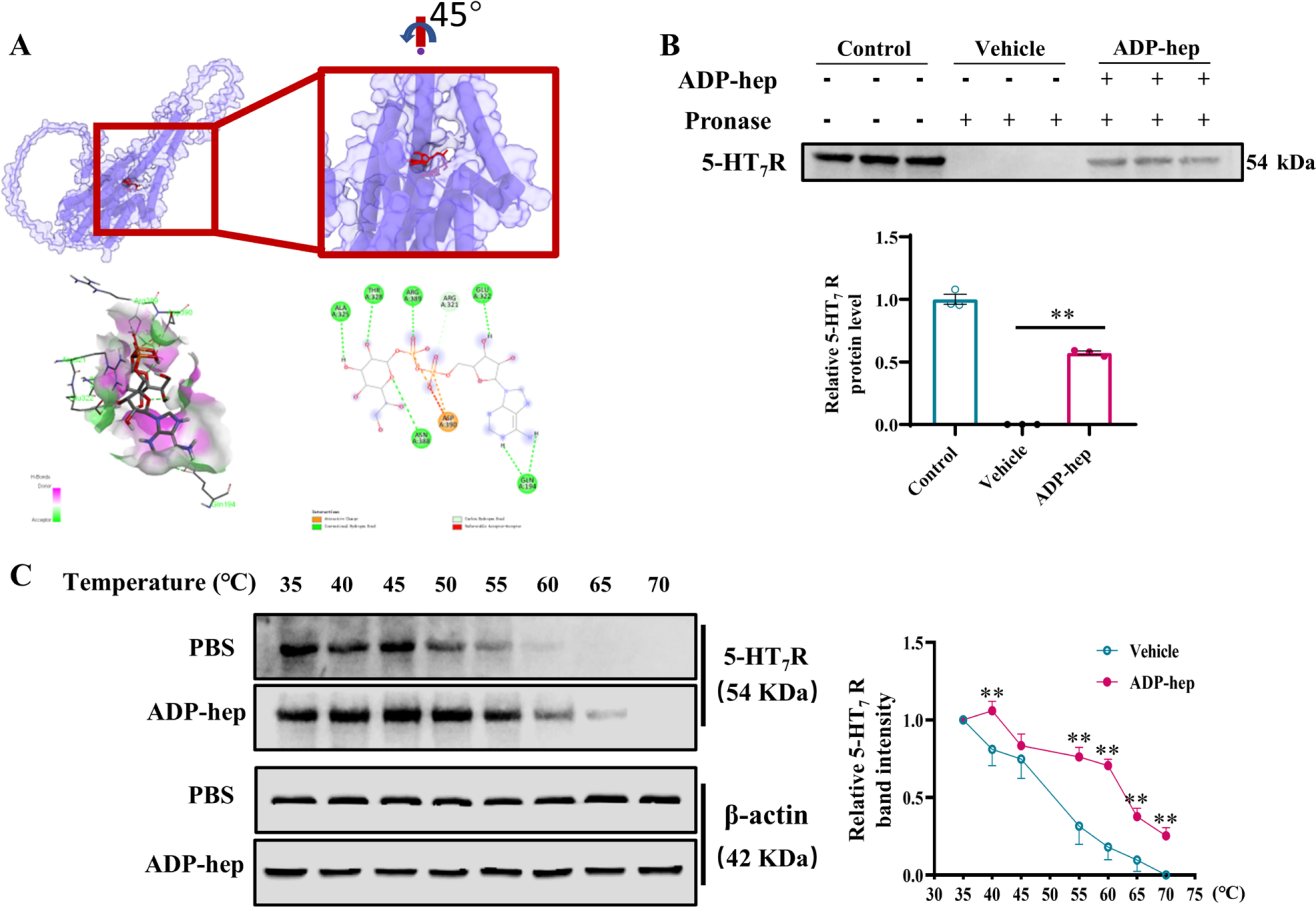
ADP‐hep may bind directly to 5‐HT_7_R in primary microglia of mice. (A) Overview on 3D structure of ADP‐hep‐5‐HT_7_R complex, and interaction between amino acid residues of ADP‐hep and 5‐HT_7_R. DARTS (B) and CETSA (C) assays confirmed the binding of ADP‐hep to 5‐HT_7_R in primary microglia cells, and β‐actin was used as the internal control, the ratio of 5‐HT_7_R to β‐actin were analyzed. The data were expressed as mean ± SEM. Data were analyzed with two‐tailed Student's *t*‐test (B). Data were analyzed with one‐way ANOVA followed by *LSD* or *Tamhani T2* post hoc test (C) (*n* = 3/group for B, *n* = 4/group for C). **p < 0.01 vs. vehicle.

In order to further explore the interaction between ADP‐hep and 5‐HT_7_R, we conducted molecular docking experiments, and the results showed that the binding energy of ADP‐hep was −7.5 kcal/mol. ADP‐hep was predicted to form hydrogen bonds with GLN194, ARG321, GLU322, THR328, ALA325, ASN388, and ARG389 of 5‐HT_7_R as well as an electrostatic interaction with ASP39 (Figure 2A). Moreover, DARTS showed that ADP‐hep inhibited 5‐HT_7_R degradation induced by pronase (Figure 2B). Meanwhile, CETSA showed that ADP‐hep significantly protected 5‐HT_7_R from temperature‐dependent denaturation (Figure 2C), suggesting that ADP‐hep may have directly interacted with 5‐HT_7_R.

### 3.3 5‐HT_7_R Deficiency Alleviated Neuronal Damage and Cognitive Impairment Induced by ADP‐Hep in Mice

**FIGURE 3:**
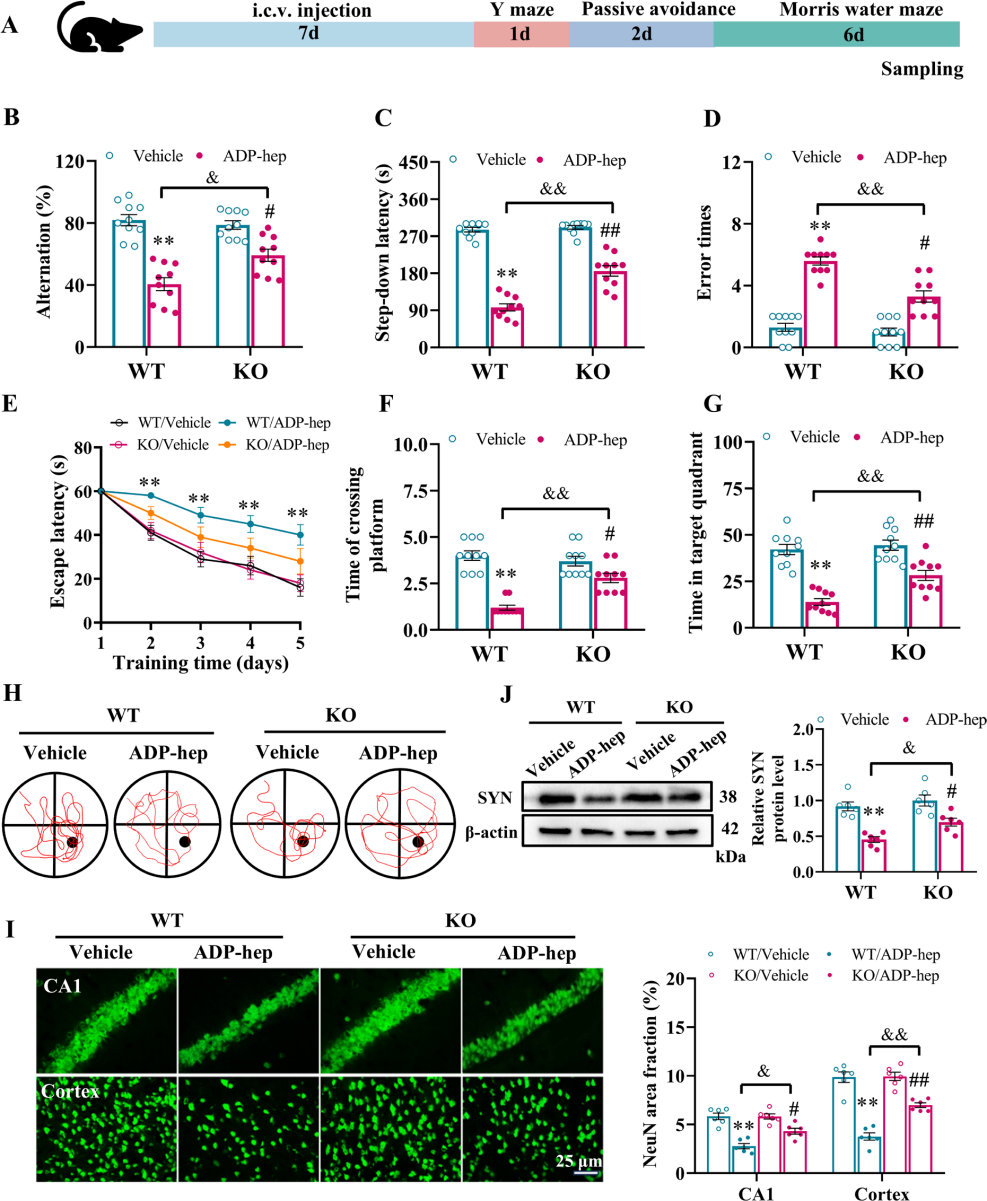
5‐HT_7_R Deficiency alleviated neuronal damage and cognitive impairment in mice treated with ADP‐hep. (A) Timeline of neurobehavioral evaluation experiments in ADP‐hep‐treated mice. (B) The effect of 5‐HT_7_R knockout on the alternation rate of ADP‐hep‐treated mice in the Y‐maze experiment. The effect of 5‐HT_7_R knockout on the step‐down latency (C) and the number of errors (D) of ADP‐hep‐treated mice in the passive avoidance experiment. The effects of 5‐HT_7_R knockout on the escape latency (E), number of platform crossings (F) and time in target quadrant (G) of ADP‐hep‐treated mice in the MWM experiment. (H) Typical path map of spatial exploration in ADP‐hep‐treated mice in the MWM experiment. (I) The expression of SYN in brain tissue in different groups of mice. (J) Immunofluorescence staining images and quantitative analysis of NeuN‐positive neurons in the hippocampal CA1 area and cortex in the brain, the ratio of SYN to β‐actin were analyzed. The data were expressed as mean ± SEM (*n* = 10/group for B–H, *n* = 6/group for I, J). Data were analyzed with two‐way ANOVA followed by the Bonferroni–Holm post hoc test (B–D, F–I). Data were analyzed with one‐way ANOVA followed by *LSD* or *Tamhani T2* post hoc test (E). *p < 0.05, **p < 0.01 vs. WT/vehicle group, ^#^ *p* < 0.05, ^##^ *p* < 0.01 vs. KO/ADP‐hep, ^&^ *p* < 0.05, ^&&^ *p* < 0.01 vs. KO/ADP‐hep.

We constructed 5‐HT_7_R knockout mice using Crispr‐Cas9 gene editing technology, and then identified mouse genotypes using agarose gel electrophoresis (Figure S4). Then, we explored the effect of ADP‐Hep on the cognitive function of 5‐HT_7_R knockout mice in vivo. The experimental procedure is shown in Figure 3A. The results of the Y‐maze experiment showed that compared to the WT/Vehicle group, the alternation rate of mice in the WT/ADP‐hep and KO/ADP‐hep groups was significantly reduced, suggesting that the short‐term memory ability of mice could be decreased after the intervention of ADP‐hep. Compared with the WT/ADP‐hep group, the alternation rate of mice in the KO/ADP‐hep group was increased, suggesting that 5‐HT_7_R knockout could partially improve the ADP‐hep‐induced short‐term memory ability of mice (Figure 3B). Similarly, the results of the jump stage experiment also showed that the step‐down latency of mice could be significantly shortened and the number of errors significantly increased after the intervention of ADP‐hep, and 5‐HT_7_R knockout could reverse the long‐term learning and memory decline induced by ADP‐hep in mice (Figure 3C,D). Furthermore, Morris water maze experiment results showed that, compared with vehicle, the escape latency of ADP‐hep‐induced mice was significantly prolonged during the training period, indicating that the learning ability of ADP‐hep‐induced mice was significantly reduced, and 5‐HT_7_R knockout could significantly reduce the escape latency of ADP‐hep‐induced mice (Figure 3E). It is suggested that inhibiting 5‐HT_7_R can significantly improve the learning disability of ADP‐hep‐induced mice. Coincidentally, we observed the spatial memory ability of the mice after they were removed from the platform. Compared with vehicle, ADP‐hep‐induced mice accurately crossed the location of the platform and the effective quadrant rate was significantly reduced, indicating that ADP‐hep‐induced mice have a significant decline in memory of the spatial location of the safe platform, and 5‐HT_7_R knockout could reverse the above abnormal changes (Figure 3F,G). The results of Figure 3H visually demonstrated the typical path map of spatial exploration of mice in each group, indicating that inhibition of 5‐HT_7_R significantly improved the spatial memory impairment in ADP‐hep‐induced mice.

Cognitive dysfunction can cause morphological changes and dysfunction of neurons, and neuron injury is one of the main indicators. In the CNS, neurons in the cortex and CA1 region of the hippocampus are the most sensitive to external stimuli [29, 30]. Therefore, we further explored the inhibitory effect of 5‐HT_7_R on ADP‐hep‐induced neuronal damage in mice. Compared with the vehicle, the number of neurons in the cortex and CA1 region of ADP‐hep‐induced mice was significantly reduced, suggesting that ADP‐hep‐induced mice had serious neuron loss. 5‐HT_7_R knockout significantly reversed neuronal loss induced by ADP‐hep (Figure 3I). In addition, synapsin (SYN) is a crucial protein within synapses, playing a pivotal role in mediating information transmission between neuronal cells [31]. Our results showed that compared with the vehicle, the expression level of SYN in the brain tissue of ADP‐hep‐induced mice was significantly reduced, indicating damage to brain synapses in ADP‐hep‐induced mice. 5‐HT_7_R knockout significantly reversed the ADP‐hep‐induced decrease in the expression of SYN (Figure 3J). These results indicated that inhibition of 5‐HT_7_R effectively improved the neuronal damage in ADP‐hep‐induced mice and promoted the increase of the number of neurons in the cortex and CA1 region of thehippocampus.

### 3.4 5‐HT_7_R Deficiency Attenuated ADP‐Hep‐Induced Ferroptosis via the p53/xCT/GPX4 Pathway

**FIGURE 4:**
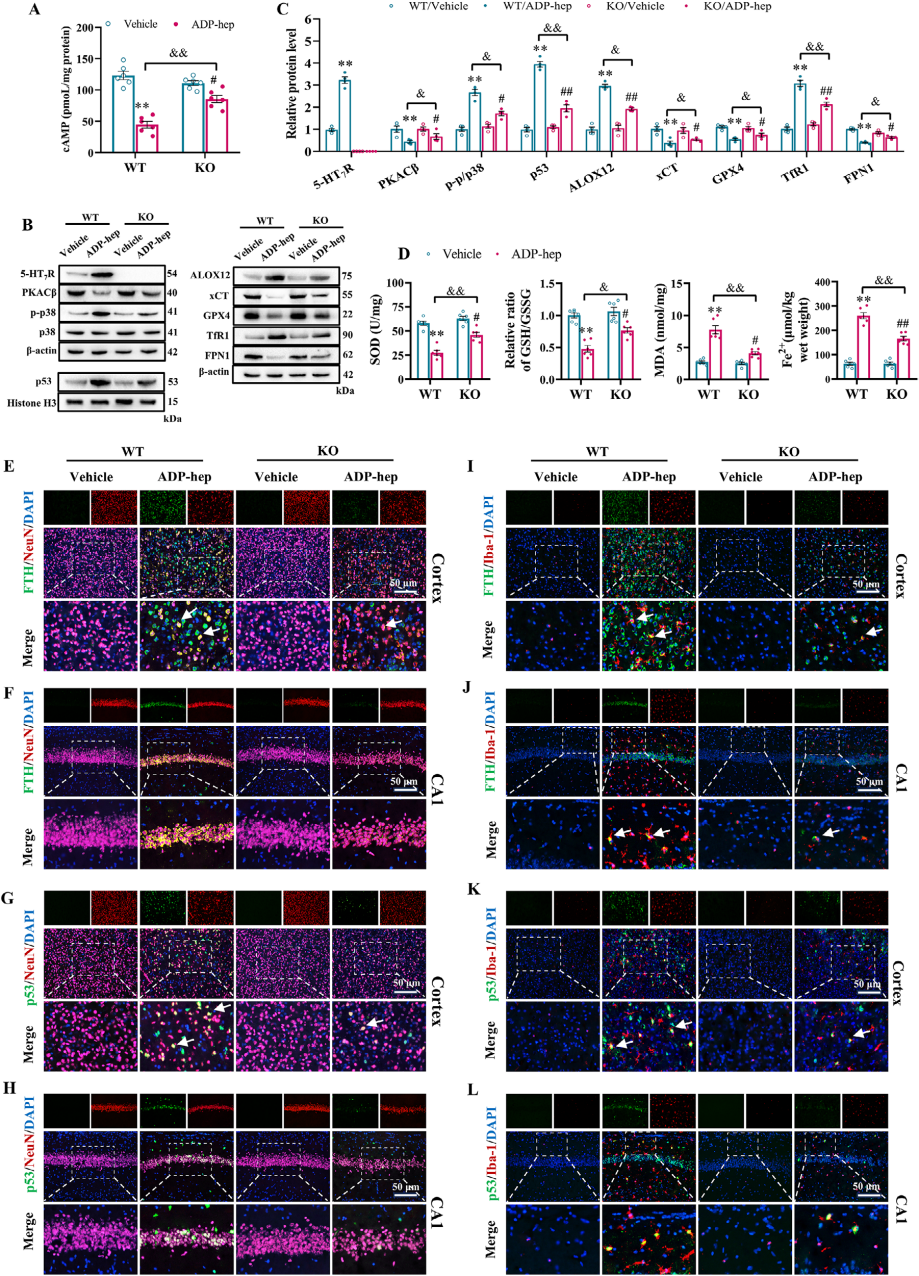
5‐HT_7_R Deficiency attenuated ferroptosis in the brain of ADP‐hep‐treated mice by regulating p53/xCT/GPX4 signaling pathway. (A) Bar graphs show the levels of cAMP in the brain. (B) Representative image of immunoblotting assays evaluating protein expression of 5‐HT_7_R, PKACß, p‐p38, p38, p53, ALOX12, xCT, TfR1, FPN1, and GPX4 in different groups of mice brain tissue, and quantification results of the immunoblotting assay (C). Analyses included the ratios of 5‐HT_7_R, PKACβ, ALOX12, xCT, GPX4, TfR1, and FPN1 to β‐actin, p‐p38 to p38, and p53 to Histone H3. (D) The relative activity of SOD, the ratio of GSH/GSSG and the levels of MDA and Fe^2+^ in brain tissue of mice in each group. (E, F) Representative immunofluorescence images of FTH and NeuN in the cerebral cortex or hippocampal CA1 subregions. (G, H) Representative immunofluorescence images of p53 and NeuN in the cerebral cortex or hippocampal CA1 subregions. (I, J) Representative immunofluorescence images of FTH and Iba‐1 in the cerebral cortex or hippocampal CA1 subregions. (K, L) Representative immunofluorescence images of p53 and Iba‐1 in the cerebral cortex or hippocampal CA1 subregions. Data were expressed as mean ± SEM (*n* = 6/group for A, D; *n* = 6/group for B, C, E, L). Data were analyzed with two‐way ANOVA followed by the Bonferroni–Holm post hoc test. *p < 0.05, **p < 0.01 vs. WT/vehicle group, ^#^ *p* < 0.05, ^##^ *p* < 0.01 vs. KO/ADP‐hep, ^&^ *p* < 0.05, ^&&^ *p* < 0.01 vs. KO/ADP‐hep.

Next, we further explored the mechanism of 5‐HT_7_R on ADP‐hep‐induced cognitive dysfunction in mice. We verified our hypothesis in vitro and in *vivo*. In vivo, the expression level of cAMP in the brain tissues of mice in each group was detected by ELISA, and the results showed that 5‐HT_7_R knockout could reverse the ADP‐hep‐induced reduction of cAMP level in the brain of mice (Figure 4A). Then, we detected the expression levels of 5‐HT_7_R and its downstream signaling pathways in mice brain tissue (Figure 4B,C). The results showed that compared with the WT/vehicle group, ADP‐hep significantly up‐regulated the expression level of 5‐HT_7_R and its downstream p38 MAPK signaling pathway, thereby regulating the expression of p53/xCT/GPX4 signaling pathway and promoting ferroptosis. 5‐HT_7_R knockout can significantly reverse the above ADP‐hep‐induced abnormal changes (Figure 4B,C). The above clues preliminarily suggest that ADP‐hep can promote ferroptosis and neuroinflammation by acting on 5‐HT_7_R and regulating its downstream signaling pathway. Moreover, studies have shown that the main features of ferroptosis include iron deposition, lipid peroxidation, and decreased antioxidant function, which are closely related to oxidative stress damage and pathophysiological mechanisms in cognitive dysfunction [32]. Therefore, biochemical kits were used to detect ferroptosis‐related indicators in the brain tissues of mice in each group. The results showed that compared with vehicle, the levels of Fe^2+^ and MDA in the brain tissue of mice induced by ADP‐hep were significantly increased, while the activity of SOD and the ratio of GSH/GSSG were significantly decreased, and the above abnormal changes could be significantly reversed by 5‐HT_7_R knockout (Figure 4D). Based on the aforementioned results, we further investigated the co‐expression of 5‐HT_7_R with neurons and microglia in the brain using immunofluorescence staining. The results demonstrated that, compared to the vehicle, ADP‐hep significantly activated microglia and up‐regulated the expression of 5‐HT_7_R in both neurons and microglial cells (Figures S5 and S6). We further observed the effect of 5‐HT_7_R knockout on ADP‐hep‐induced ferroptosis of neurons and microglia in the mouse brain. Studies have shown that ferritin and p53 are commonly used to characterize ferroptosis [32, 33]. On this basis, our immunofluorescence results showed that compared with vehicle, ADP‐hep induced the expression of ferritin and p53 in neurons and microglia in the cortex and CA1 region of the mice brain to be significantly up‐regulated, suggesting that ADP‐hep intervention can promote ferroptosis in neurons (Figures 4G,H and S7A,B) and microglia (Figures 4I–L and S7C,D) of the mice brain. 5‐HT_7_R knockout can significantly reverse the abnormal changes induced by ADP‐hep. The above results indicated that 5‐HT_7_R inhibition can attenuate ADP‐hep‐induced ferroptosis in mice neurons and microglia by regulating its downstream signaling.

**FIGURE 5:**
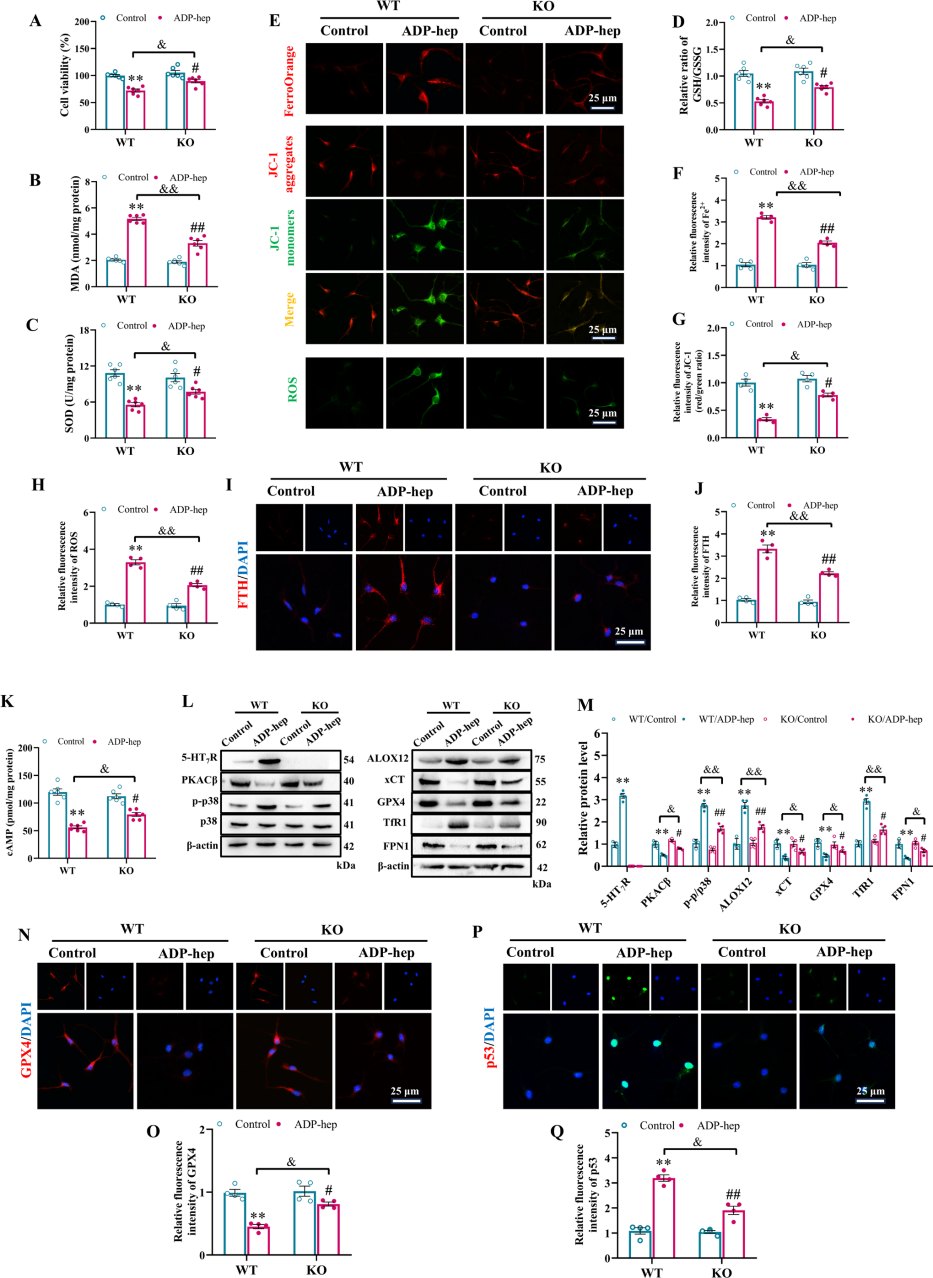
5‐HT_7_R Deficiency inhibited ADP‐hep‐induced neuronal ferroptosis in primary neuronal cells via the p53/xCT/GPX4 pathway. (A) The effect of different stimulation schemes on the survival rate of primary neuronal cells. The levels of MDA (B), the relative activity of SOD (C), the ratio of GSH/GSSG (D) and in the primary neuronal cells. (E, H) Representative immunofluorescence images and quantitative analysis of Fe^2+^, JC‐1 staining, and ROS staining in primary neuronal cells. (I, J) Representative immunofluorescence images and quantitative analysis of FTH staining in primary neuronal cells. (K) Bar graphs show the levels of cAMP in the primary neuronal cells. (L) Representative image of immunoblotting assays evaluating protein expression of 5‐HT_7_R, PKACβ, p‐p38, p38, p53, ALOX12, xCT, GPX4, TfR1, and FPN1 in primary neuronal cells, and quantification results of the immunoblotting assay (M). Analyses included the ratios of 5‐HT_7_R, PKACβ, ALOX12, xCT, GPX4, TfR1, and FPN1 to β‐actin, p‐p38 to p38. (N, O) Representative immunofluorescence images and quantitative analysis of GPX4 in primary neuronal cells. (P, Q) Representative immunofluorescence images and quantitative analysis of p53 in primary neuronal cells. Data were expressed as mean ± SEM. (*n* = 6/group for A‐D, K; *n* = 4/group for E–J, L–P). Data were analyzed with followed by the Bonferroni–Holm post hoc test. **p < 0.01 vs. WT/vehicle group, ^#^ *p* < 0.05, ^##^ *p* < 0.01 vs. KO/ADP‐hep, ^&^ *p* < 0.05, ^&&^ *p* < 0.01 vs. KO/ADP‐hep.

**FIGURE 6:**
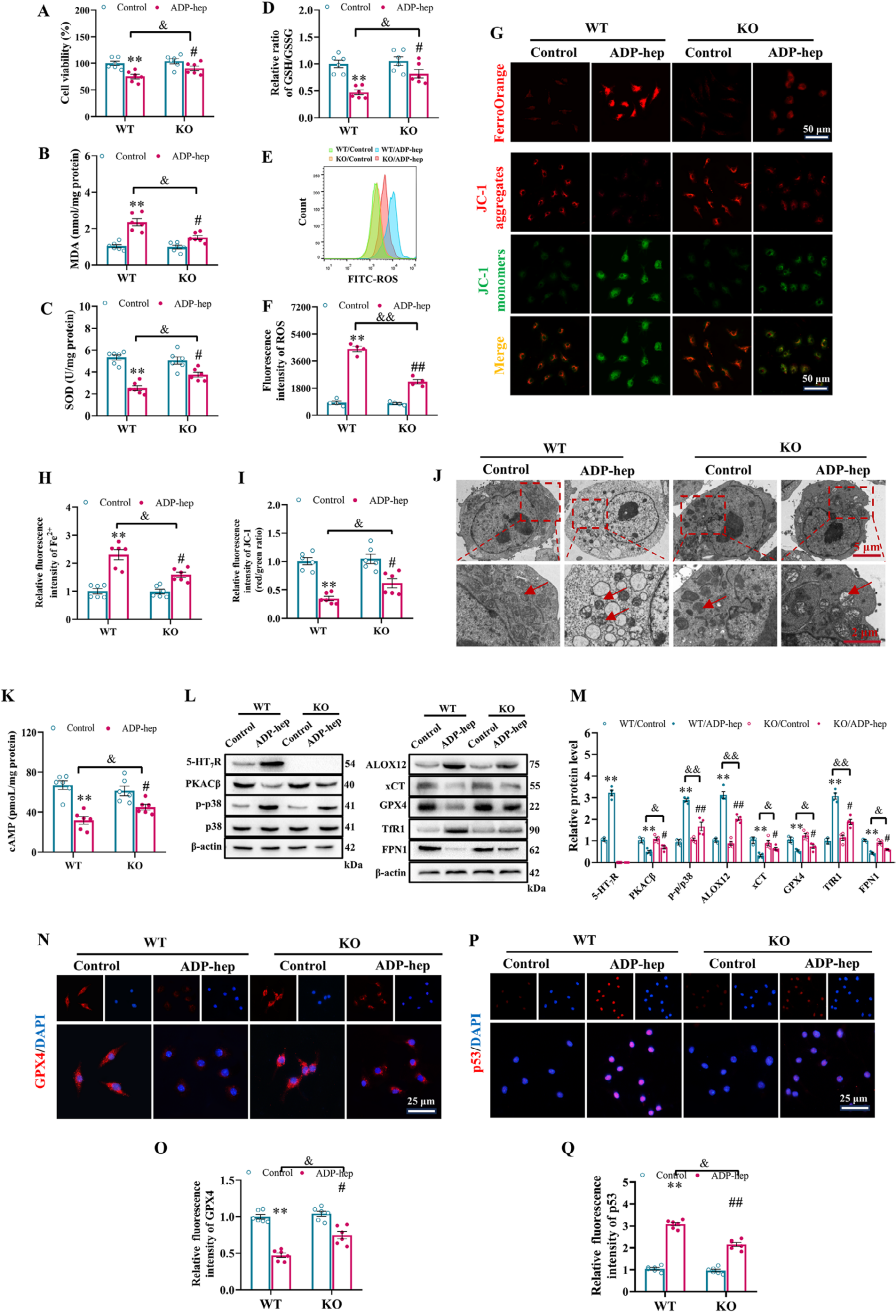
5‐HT_7_R Deficiency inhibited ADP‐hep‐induced microglial ferroptosis in primary microglial cells via the p53/xCT/GPX4 pathway. (A) The effect of different stimulation schemes on the survival rate of microglial cells. The levels of MDA (B), the relative activity of SOD (C), the ratio of GSH/GSSG (D) and in the microglia cells. (E, F) The intracellular ROS levels were examined by flow cytometry, and quantification was analyzed by Image J software. (G, I) Representative immunofluorescence images and quantitative analysis of Fe^2+^ and JC‐1 staining in primary microglia. (J) TEM observations of mitochondrial ultrastructure in microglia cells. (K) Bar graphs show the levels of cAMP in the primary microglial cells. (L) Representative image of immunoblotting assays evaluating protein expression of 5‐HT_7_R, PKACβ, p‐p38, p38, p53, ALOX12, xCT, GPX4, TfR1, and FPN1 in primary microglial cells, and quantification results of the immunoblotting assay (M). Analyses included the ratios of 5‐HT_7_R, PKACβ, ALOX12, xCT, GPX4, TfR1, and FPN1 to β‐actin, p‐p38 to p38. (N, O) Representative immunofluorescence images and quantitative analysis of GPX4 in primary microglia. (P, Q) Representative immunofluorescence images and quantitative analysis of p53 in primary microglia. Data were expressed as mean ± SEM. (*n* = 6/group for A–D, G, I, K, N–Q; *n* = 4/group for E–F, L–M). Data were analyzed with two‐way ANOVA followed by the Bonferroni–Holm post hoc test. **p < 0.01 vs. WT/vehicle group, ^#^ *p* < 0.05, ^##^ *p* < 0.01 vs. KO/ADP‐hep, ^&^ *p* < 0.05, ^&&^ *p* < 0.01 vs. KO/ADP‐hep.

Based on the above results, we further demonstrated in vitro that ADP‐hep can significantly up‐regulate the expression of 5‐HT_7_R in primary neurons (Figures S8 and S9). Meanwhile, we investigated the relationship between the dynamic polarization states of microglia and ferroptosis. The results demonstrated that ADP‐heptose‐induced ferroptosis in M2‐type microglia was most pronounced at the 24 h (Figure S10). Consequently, the 24 h time point was consistently adopted for all subsequent experiments. Subsequently, we further explored the mechanism of 5‐HT_7_R on ADP‐hep‐induced ferroptosis of primary neurons and primary microglia in vitro. We found that 5‐HT_7_R deficiency significantly reversed the decreased activity of primary neurons and primary microglia due to ADP‐hep stimulation (Figures 5A and 6A). Furthermore, the deficiency of 5‐HT_7_R can significantly reverse the ADP‐hep induction leading to the downregulation of SOD activity and the ratio of GSH/GSSG, as well as the high level of MDA (Figures 5B–D and 6B–D). These clues suggest that 5‐HT_7_R deficiency can inhibit the abnormal changes of primary neurons and primary microglia caused by ferroptosis. Additionally, we examined the effects of 5‐HT_7_R on oxidative stress levels. The results showed that 5‐HT_7_R deficiency reversed the production of Fe^2+^ and ROS and the reduction of mitochondrial membrane potential (MMP) in primary neurons and primary microglia treated with ADP‐hep (Figures 5E–H and 6E–I). The characteristics of ferroptosis in primary neurons (Figure 5I,J) and primary microglia (Figure 6J) were observed by immunofluorescence and transmission electron microscopy (TEM), respectively. These clues suggest that 5‐HT_7_R deficiency can inhibit the abnormal changes of primary neurons and primary microglia caused by ferroptosis induced by ADP‐hep. Moreover, we also examined the effects of 5‐HT_7_R deficiency on the expression levels of 5‐HT_7_R and its downstream p38 MAPK signaling pathway, as well as the key ferroptosis‐related signaling pathway p53/xCT/GPX4, in ADP‐hep‐treated cells in vitro. The results were largely consistent with those obtained from the in vivo experiments (Figures 5K–M and 6K–M). Our immunofluorescence findings corroborated the western blot results (Figures 5N–Q and 6N–Q). These lines of evidence suggest that inhibition of 5‐HT_7_R can attenuate ADP‐hep‐induced ferroptosis mediated by the p53/xCT/GPX4 signaling pathway in primary neurons and primary microglia through modulation of its downstream signaling.

### 3.5 5‐HT_7_R Deficiency Alleviated ADP‐Hep‐Induced Inflammation and Neuronal Injury via Inhibiting M2 Microglial Ferroptosis

**FIGURE 7:**
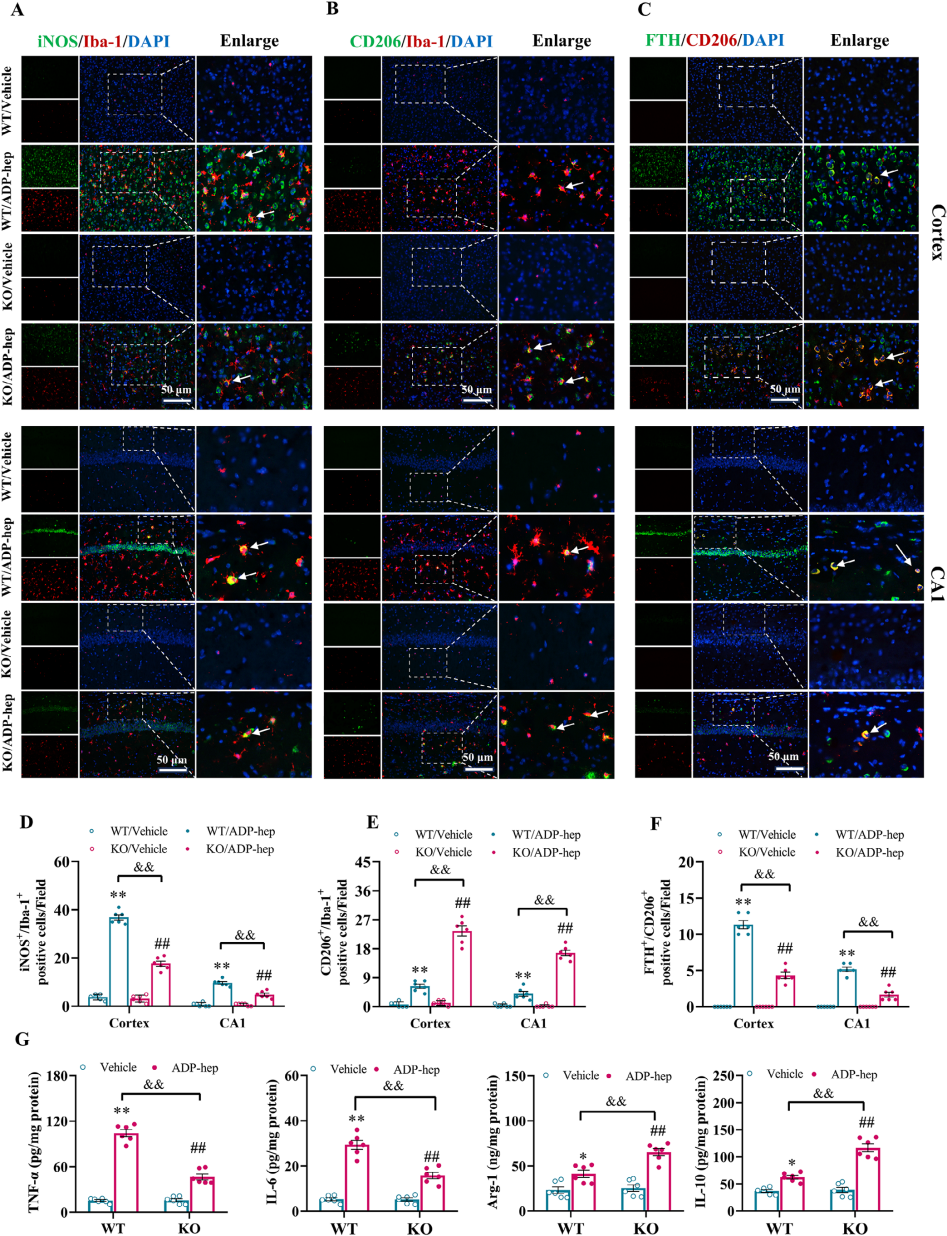
5‐HT_7_R deficiency improved abnormal polarization of microglia and neuroinflammation in the brain of ADP‐hep‐treated mice. (A, B) Double immunofluorescence staining of iNOS and Iba1 in the cerebral cortex and hippocampus CA1 subregions. (C, D) Double immunofluorescence staining of CD206 and Iba1 in the cerebral cortex and hippocampus CA1 subregions. (E, F) Double immunofluorescence staining of CD206 and Ferrtin in the cerebral cortex and hippocampus CA1 subregions. (G) The levels of TNF‐α, IL‐6, IL‐10 and Arg‐1 in the brain detected by ELISA. The data were expressed as mean ± SEM (*n* = 6/group). Data were analyzed with two‐way ANOVA followed by the Bonferroni–Holm post hoc test. *p < 0.05, **p < 0.01 vs. WT/vehicle group, ^#^ *p* < 0.05, ^##^ *p* < 0.01 vs. KO/ADP‐hep, ^&^ *p* < 0.05, ^&&^ *p* < 0.01 vs. KO/ADP‐hep.

Microglia, as the first line of immune defense in the brain, regulate inflammatory responses and maintain immune inflammatory microenvironment homeostasis by activating into M1/M2 phenotype [34]. We observed the co‐expression of Iba‐1 with markers of different sub‐types of microglia by immunofluorescence. The results showed that compared with the WT/vehicle group, the iNOS/Iba‐1 and CD206/Iba‐1 positive area ratio of hippocampus CA1 and cortex in the WT/ADP‐hep group were significantly increased, indicating that ADP‐hep can induce microglial over‐activation. 5‐HT_7_R knockout can significantly reverse the abnormal polarization of microglia induced by ADP‐hep, as shown by significantly decreased iNOS/Iba‐1 double positive area ratio and significantly increased CD206/Iba‐1 double positive area ratio (Figure 7A–D). At the same time, we also focused on the co‐expression of M2 microglia and ferritin, and the results showed that compared with the WT/Vehicle group, the positive area ratio of Ferritin/CD206 in the WT/ADP‐hep group was significantly increased, and 5‐HT_7_R knockout could significantly reverse the above abnormal changes. It is suggested that 5‐HT_7_R knockout can inhibit iron deposition in M2 microglia induced by ADP‐hep (Figure 7E,F). Correspondingly, the signature cytokines of M1/M2 microglia in mice brain tissues also underwent similar changes along with the change of microglia polarization (Figure 7G). Additionally, from a protein‐level perspective, we investigated the effects of cAMP analogs or 5‐HT_7_R agonists/inhibitors on ADP‐hep‐induced ferroptosis in M2 microglia. The results were consistent with the aforementioned findings (Figure S11). These clues suggest that 5‐HT_7_R knockout can inhibit the iron death of M2 microglia induced by ADP‐hep, then inhibit the abnormal polarization of microglia, and thus inhibit neuroinflammation.

**FIGURE 8:**
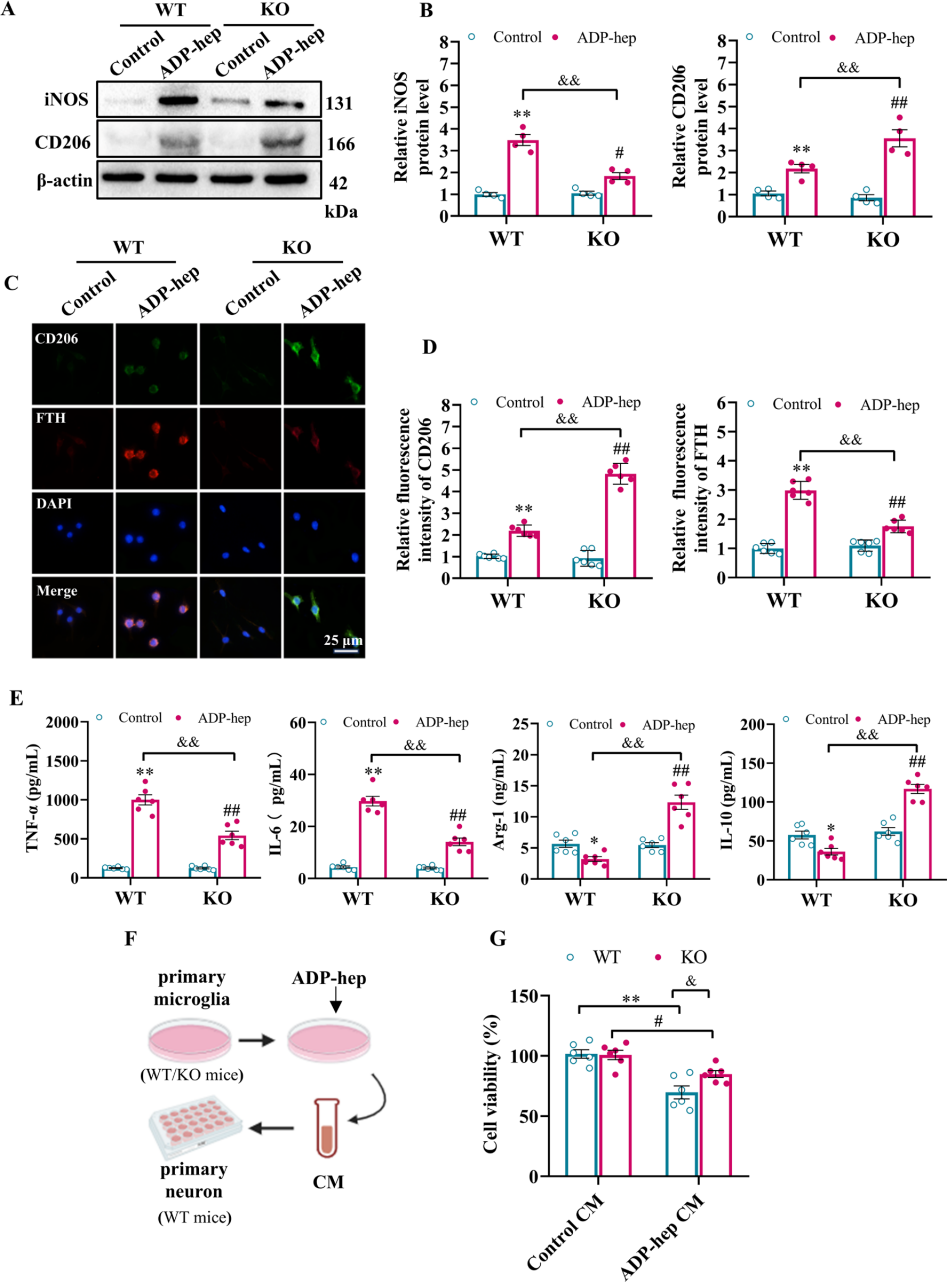
5‐HT_7_R Deficiency alleviated ADP‐hep‐induced inflammation and neuronal injury via inhibiting M2 microglial ferroptosis in vitro. (A, B) Representative immunoblots and quantitative analysis of iNOS, CD206 in primary microglia cells. The ratio of iNOS and CD206 to β‐actin were analyzed. (C, D) Double immunofluorescence staining images and quantitative analysis of CD206 and Ferrtin in microglial cells. (E) The levels of TNF‐α, IL‐6, IL‐10 and Arg‐1 in the supernatants of primary microglia cells detected by ELISA. (F) Schematic representation of the experimental setup, where primary neurons are stimulated with conditioned medium (CM) derived from ADP‐hep‐treated primary microglia cells from WT and KO mice. (G) A bar chart shows the cell viability of primary neurons. The data were expressed as mean ± SEM (*n* = 4/group for A, B, *n* = 6/group for D–G). Data were analyzed with two‐way ANOVA followed by the Bonferroni–Holm post hoc test. *p < 0.05, **p < 0.01 vs. WT/control group, ^#^ *p* < 0.05, ^##^ *p* < 0.01 vs. KO/ADP‐hep, ^&^ *p* < 0.05, ^&&^ *p* < 0.01 vs. KO/ADP‐hep.

The previous results have shown that microglia undergo ferroptosis after ADP‐hep intervention. However, previous studies have shown that different subtypes of microglia have different sensitivities to ferroptosis, among which M1 microglia are insensitive to ferroptosis, while M2 microglia are highly sensitive to ferroptosis [35]. In view of the phenomenon of rapid decline of M2 microglia in the acute stage of cognitive impairment pathology, it may be related to the high sensitivity of M2 microglia to ferroptosis. Therefore, in the following in vitro, we focused on the effect of 5‐HT_7_R knockout on ferroptosis of M2 microglia induced by ADP‐hep. The results showed that, similar to the results in vivo, the expression of iNOS and CD206 was up‐regulated to varying degrees after ADP‐hep treatment compared with control. 5‐HT_7_R knockout can significantly down‐regulate the overexpression of iNOS caused by ADP‐hep, but can further up‐regulate the expression of CD206 (Figure 8A,B). In addition, immunofluorescence results showed that compared with 5‐HT_7_R^WT^, 5‐HT_7_R knockout could significantly up‐regulate the fluorescence intensity of CD206, while inhibiting the fluorescence intensity of ferritin (Figure 8C,D). Correspondingly, the characteristic cytokines of M1/M2 microglia in the cell culture supernatant also underwent similar changes along with polarization changes in microglia (Figure 8E). These clues suggest that 5‐HT_7_R knockout can play a neuroprotective role by inhibiting ferroptosis of M2 microglia induced by ADP‐hep, slowing down the decline in the proportion of M2 microglia, and then alleviating the inflammatory microenvironment. It is worth mentioning that we treated primary neuronal cells with CM of primary microglia and found that 5‐HT_7_R knockout inhibited the ADP‐hep‐induced microglia inflammatory microenvironment and further affected neuronal survival (Figure 8F,G). Previous studies have shown that there is interaction between microglia and neurons, and the inflammatory microenvironment constructed by microglia can significantly affect the survival of neurons [36], which is consistent with our conclusion. In conclusion, inhibition of 5‐HT_7_R can inhibit ferroptosis of neuronal and M2 microglia induced by ADP‐hep through the p53/xCT/GPX4 signaling pathway, improve the inflammatory microenvironment, and then affect the survival of neurons, exerting neuroprotective effects. It is suggested that 5‐HT_7_R may become a new target for the prevention and treatment of neurodegenerative diseases, and provide a new idea and theoretical basis for the subsequent development of corresponding drugs for the treatment of neurodegenerative diseases.

## 4 Discussion

Neuroinflammation, an important characteristic of the pathogenesis and progression of various neurodegenerative diseases, is triggered by a series of signals such as infection, trauma, toxic metabolites, and autoimmune reactions, which can impair neuronal structure and function, potentially leading to neuronal death. Neuroinflammation is intricately linked to cognitive deficits associated with a variety of diseases, thus making its inhibition regarded as a promising therapeutic strategy for disorders related to cognitive impairment [37]. The pathogenesis of LPS‐induced cognitive impairment likely involves neuroinflammation, oxidative stress, blood–brain barrier disruption, and neuronal damage, with neuroinflammation being central to the onset and progression of cognitive deficits [38]. Numerous studies have confirmed that LPS can induce neurobehavioral deficits, including reduced learning and memory capabilities, diminished motor complexity, heightened anxiety, and depression‐like behaviors [39]. Furthermore, intraperitoneal injection of LPS serves as a mouse model for studying neuroinflammation‐induced cognitive impairment [40]. ADP‐hep, a soluble intermediate in the LPS biosynthetic pathway, has been demonstrated to induce inflammation through induction of ALPK1 activation [18]. In order to explore whether ADP‐hep, like LPS, can also induce neuroinflammation and subsequent cognitive impairment, we administered ADP‐hep into the lateral ventricle of mice (i.c.v. once daily for 7 days) to provoke cognitive impairment, which was validated through neurobehavioral assessments in vivo (Figure 3).

It is intriguing that ADP‐hep, an endogenous PAMP, might interact with PRRs other than ALPK1, yet no studies have addressed this possibility. As the primary immune cells in the CNS, microglia are the principal source of inflammatory mediators, and their overactivation‐induced neuroinflammation is identified as one of the important pathological mechanisms in the development of numerous neurological disorders [28]. It is widely accepted that LPS can stimulate the abnormal activation of microglia, either directly or indirectly. Against this backdrop, we isolated primary microglia from the brains of mice with ADP‐hep‐induced neuroinflammation and, through mRNA sequencing analysis, identified 5‐HT_7_R as a potential PRR for ADP‐hep (Figure 1). As the latest discovered member of the 5‐HT receptor family, 5‐HT_7_R has emerged as one of the important targets for research and development of new antidepressant drugs [41]. 5‐HT_7_R plays a pivotal role in the treatment of cognitive impairments induced by various factors. Studies have demonstrated that 5‐HT7 receptor antagonists, lurasidone and vortioxetine, can improve global cognitive function in clinical settings [42, 43]. Furthermore, 5‐HT7 receptor antagonists (SB‐269970, SB‐656104‐A) have been shown to ameliorate cognitive impairment induced by various etiologies [44, 45]. These findings are consistent with our results. However, the 5‐HT7R exhibits bidirectional regulatory effects: on one hand, as a G protein‐coupled receptor, it activates the Gs/cAMP/PKA pathway to enhance cognitive function. On the other hand, under conditions of hyperactive serotonergic tone or neuroinflammation, 5‐HT7R may exacerbate neuronal damage through pro‐inflammatory pathways such as β‐arrestin/NF‐κB, where antagonists can improve cognition by suppressing aberrant signaling [46]. For instance, 5‐HT7R agonists, such as LP‐211 and AS‐19, have been shown to reverse Aβ‐induced neuronal damage and cognitive impairment in AD animal models [47, 48]. Additionally, 5‐HT7R has been shown to be specifically expressed on regulatory T cells (Tregs) in the brain and is essential for their function. Tregs accumulate in the brain following ischemic stroke, contributing to neuroprotection and recovery [49]. Htr7+ Tregs can alleviate neuroinflammation and prevent neuronal damage by inhibiting CD8 T cell infiltration into the brain and suppressing excessive microglial activation, thereby improving LPS‐induced cognitive impairment in mice [50]. Therefore, activation or inhibition of 5‐HT_7_R may improve cognitive impairment under different conditions, and the key is to select appropriate intervention strategies based on specific pathological mechanisms. Future studies need to further clarify the mechanism of action of 5‐HT_7_R in different disease models to guide precise treatment. It has been reported that this receptor may affect neuronal plasticity and activation of glial cells, and is involved in the regulation of vital signal transduction pathways in the CNS, playing a crucial role in the occurrence and development of neurological diseases [47]. By stimulating Gαs protein to activate adenylate cyclase, 5‐HT_7_R can induce intracellular accumulation of cAMP, which in turn activates PKA by binding to its regulatory subunits, thus promoting the formation of synaptic connections [51, 52]. As one of the downstream effectors of 5‐HT_7_R, p38 is instrumental in protecting cerebral vasculature and modulating memory and cognitive functions [53]. We further investigated the regulatory effects of ADP‐hep on 5‐HT7R using molecular docking, DARTs, and CETSA in vitro. The present results indicate a potential direct interaction between ADP‐hep and 5‐HT7R (Figure 2). Numerous studies have indicated that the 5‐HT_7_R, as a GPCR, is not stable [54, 55]. It is generally difficult to detect the direct interaction between ligands and the full‐length sequence of 5‐HT_7_R using surface plasmon resonance (SPR) and other methods. The interaction between the 5‐HT_7_R and ligand needs to be studied by designing full‐length sequences or active fragments of 5‐HT_7_R with fusion proteins or heat stability mutations. Recently, it was found that the binding site of 5‐carboxamidotryptamine (5‐CT) and 5‐HT_7_R was predicted, mainly binding to the transmembrane region of 5‐HT_7_R, and the binding mode of 5‐CT and 5‐HT_7_R was similar to 5‐HT (a classic natural endogenous ligand of 5‐HT_7_R) [56]. Our molecular docking results showed that the interaction sites between ADP‐hep and 5‐HT_7_R were GLN194, ARG321, GLU322, THR328, ALA325, ASN388, and ARG389. In addition, it has been found that S100B can directly interact with the intracellular segment of 5‐HT_7_R (RVEPDSVI) through peptide synthesis and SPR techniques in vitro [57]. Based on the above research literature on the interaction between ligands and specific amino acid residues of 5‐HT_7_R and the in‐depth study on the characteristics of 5‐HT7R, it is hoped to further confirm the interaction between ADP‐hep and 5‐HT_7_R and the specific binding site in the future. Furthermore, ADP‐hep could regulate 5‐HT_7_R expression and its downstream signaling pathway in vivo and *vitro*, and the aberrations induced by ADP‐hep were significantly reversed after 5‐HT_7_R was knocked out (Figures 4 and 6). Altogether, the present results suggest that ADP‐hep may precipitate cognitive impairment in mice through regulating 5‐HT_7_R‐mediated neuroinflammation. These findings provide a novel avenue for investigating cognitive impairment‐related diseases and establishing a preliminary foundation for further studies into the pathogenesis underlying such conditions in the future. It is noteworthy that in the development and progression of cognitive impairment‐related diseases, particularly neurodegenerative diseases, in addition to the neuroinflammation we focus on, 5‐HT_7_R can also play a critical role in maintaining and restoring cognitive function by regulating multiple mechanisms, including abnormal protein aggregation (such as Aβ deposition), synaptic dysfunction (such as impaired synaptic plasticity), mitochondrial dysfunction (such as oxidative stress), and dysregulation of neurotrophic factors (such as brain‐derived neurotrophic factor) [47]. Future research needs to further explore the specific mechanisms of 5‐HT_7_R in human diseases and promote its clinical translation.

Initial research posited that necrosis was the sole form of cell death capable of instigating inflammation. However, with the discovery of novel cell death modalities such as pyroptosis, necroptosis, ferroptosis, and NETosis, it has become evident that these multiple forms of cell death can activate the immune system and provoke inflammatory responses [58]. Accumulated evidence suggests that immunogenic ferroptosis contributes to inflammation in certain diseases, with some studies even implicating it as the initial trigger of inflammation prior to other cell death forms [59, 60]. Furthermore, aberrant brain iron deposition is intimately connected with cognitive impairment [61]. Studies have identified excessive iron accumulation in the brains of rats with vascular cognitive impairment due to chronic cerebral hypoperfusion, with the CA1 region exhibiting the most significant iron deposition and neuronal death, and similar findings have been confirmed in neurodegenerative diseases like AD [61]. Nervous tissues are especially susceptible to damage from excessive iron or redox reactions. Concurrently, the brain is particularly vulnerable to lipid peroxidation due to its high unsaturated fatty acids and elevated oxygen consumption rate [62]. In recent years, the involvement of p53 in ferroptosis has emerged as a new research field, with evidence indicating that p53 may act as a central regulator in both canonical and non‐canonical ferroptotic pathways [33]. Additionally, the GSH‐GPX4 antioxidant system has garnered extensive research attention. Consequently, the p53/xCT/GPX4 signaling pathway is recognized as the key regulatory axis of ferroptosis. Based on the modulation of ferroptotic markers and the p53/xCT/GPX4 pathway, our study further confirmed that 5‐HT_7_R deficiency may exert a neuroprotective effect by suppressing ferroptosis induced by ADP‐hep both in vivo (Figure 5) and in vitro (Figure 6).

Studies have shown that iron accumulation in microglia can lead to the activation of microglia, aggravate the progression of neuroinflammation, increase the production of ROS, and further iron overload, thus causing neuronal death and cognitive impairment [63]. Interestingly, the accumulation of inducible nitric oxide synthase (iNOS) in M1 microglia makes them resistant to ferroptosis, while M2 microglia lacking iNOS show a high sensitivity to pro‐ferroptosis stimulation. Therefore, the M1 phenotype is mainly manifested in brain injury, thus forming a pro‐inflammatory microenvironment in the CNS [36]. Other studies have shown that after subarachnoid hemorrhage, the sensitivity of endothelial cells and M2 cells to ferroptosis is enhanced by up‐regulating ALOX15, which destroys the blood–brain barrier, aggravates central inflammation, and further damages neurons [64]. This evidence fully reveals the correlation between ferroptosis of M2 microglia and its mediated neuroinflammation and cognitive impairment. It was suggested that inhibiting the ferroptosis of existing M2 microglia could slow down the decline in the proportion of M2 microglia and further inhibit the inflammatory microenvironment to promote the survival of neurons, which was also confirmed in this study (Figures 7 and 8). This may lead to innovative interventions for the prevention and treatment of cognitive impairment related diseases.

## 5 Conclusion

In summary, we demonstrate for the first time that 5‐HT_7_R is a potential PRR for ADP‐hep. Moreover, 5‐HT_7_R deficiency can promote the recovery of ADP‐hep‐induced cognitive deficits in mice by alleviating neuronal and M2 microglial ferroptosis, and thereby inhibiting neuroinflammation and promoting neuronal survival. These findings suggest that targeting 5‐HT_7_R is a potential therapeutic approach for cognitive impairment‐related diseases.

## Author Contributions

**Xiao Zou:** methodology, investigation, formal analysis. Yu‐Xin Yang: writing – review and editing. **Han‐Yinan Yang**, **Bing‐Jie Yue**, **YYan‐Rong Yang:** investigation. **Meng‐Yang Li:** writing – original draft. **Ou Du**, **Gan Qiao:** methodology. **YYi‐Jin Wu:** writing – original draft, writing – review and editing. **Jun‐Rong Du:** conceptualization, funding acquisition. **Fang‐Yi Long:** formal analysis, writing – review and editing. All authors reviewed the manuscript.

## Ethics Statement

The animal experiments were approved by the Animal Experimentation Ethics Committee of Sichuan University (K2024026). All care and treatment of experimental animals were in strict accordance with the guidelines of the experimental animal management requirements of Sichuan University.

## Consent

The authors have nothing to report.

## Conflicts of Interest

The authors declare no conflicts of interest.

## Supporting information


**Figure S1.** Effects of ADP‐hep on the protein expression of inflammatory factors in mice brain following ADP‐hep single intracerebroventricular administration. Protein levels of the TNF‐α, IL‐6 in the brain detected by ELISA. Data are expressed as mean ± SEM (*n* = 8/group). Data were analyzed with one‐way ANOVA followed by *LSD* or *Tamhani T2* post hoc test. *p < 0.05, **p < 0.01 vs. Vehicle.
**Figure S2.** Sorting and identification of microglia. (A, B) Schematic diagram of flow cytometry sorting of microglia from WT mice brain. (C) Immunofluorescence images show the expression of Iba‐1 for the identification of isolated cultured primary microglia from WT mice.
**Figure S3.** GO/KEGG enrichment analysis and gene set enrichment analysis in the microglia sorted from the mice brain. (A–C) GO enrichment analysis. (D, E) KEGG enrichment analysis. (F–H) Gene set enrichment analysis.
**Figure S4.** Genotyping of wild‐type (WT), 5‐HT_7_R heterozygous (5‐HT_7_R^+/−^), 5‐HT_7_R knockout (5‐HT_7_R^−/−^) mice. (A) Schematic diagram of mouse 5‐HT_7_R agarose gel electrophoresis results and (B) agarose gel electrophoresis results of part of mouse genotypes.
**Figure S5.** Representative immunofluorescence images and quantitative analysis of 5‐HT_7_R and NeuN in the cerebral cortex or hippocampal CA1 subregions in the mice following 7 consecutive days of ADP‐hep intracerebroventricular administration (A–C). Scale bar = 50 μm. The data were expressed as mean ± SEM (*n* = 4/group). Data were analyzed with two‐tailed Student’s *t*‐test. **p < 0.01 vs. Vehicle group.
**Figure S6.** Representative immunofluorescence images and quantitative analysis of 5‐HT_7_R and Iba1 in the cerebral cortex or hippocampal CA1 subregions in the mice following 7 consecutive days of ADP‐hep intracerebroventricular administration (A–C). Scale bar = 50 μm. The data were expressed as mean ± SEM. (*n* = 4/group). Data were analyzed with two‐tailed Student’s *t*‐test. **p < 0.01 vs. Vehicle group.
**Figure S7.** Quantitative analyses of co‐localization of Ferrtin and NeuN (A), p53 and NeuN (B), Ferrtin and Iba‐1 (C), p53, and Iba‐1 (D) in the cerebral cortex or hippocampal CA1 subregions. Data were expressed as mean ± SEM (*n* = 4/group). Data were analyzed with two‐way ANOVA followed by the Bonferroni–Holm post hoc test. **p < 0.01 vs. WT/vehicle group, ^##^ *p* < 0.01 vs. KO/ADP‐hep, ^&^ *p* < 0.05, ^&&^ *p* < 0.01 vs. KO/ADP‐hep.
**Figure S8.** Identification of primary neuronal cells. Immunofluorescence images show the expression of MAP‐2 for the identification of isolated cultured primary neuronal cells from WT mice. Scale bar = 50 μm.
**Figure S9.** The relative expression of 5‐HT_7_R in primary neuronal cells were detected by immunofluorescence after ADP hep (100 μM) treatment for 24 h. The data were expressed as mean ± SEM, *n* = 6/group. Data were analyzed with two‐tailed Student’s *t*‐test. **p < 0.01 vs. Control group.
**Figure S10.** Temporal dynamics of M1/M2 microglial phenotypic polarization. BV2 cells were incubated with ADP‐hep (100 μM) for 0 h, 6 h, 12 h, and 24 h. (A–C) Representative immunofluorescence images and quantitative analysis of iNOS and CD206 (400×), CD206 (green), and iNOS (red). (D, E) Representative immunofluorescence images and quantitative analysis of CD206 and FTH (400×), CD206 (green), and FTH (red). The data were expressed as mean ± SEM, *n* = 6/group. Data were analyzed with two‐tailed Student’s *t*‐test. *p < 0.05, **p < 0.01 vs. 0 h group.
**Figure S11.** Effects of 5‐HT_7_R agonist, dibutyryl cAMP, 5‐HT_7_R antagonist on ADP‐hep induced ferroptosis in BV2 cells. (A) The survival rate of BV2 cells. (B) The cAMP content, (C) the content of MDA and (D) the level of Fe^2+^ in BV2 cells. (E–I) Representative immunofluorescence images and quantitative analysis of ROS, GPX4, CD206, FTH (400×), ROS (green), GPX4 (red), CD206 (green), FTH (red), and DAPI (blue). The data were expressed as mean ± SEM, *n* = 6/group. Data were analyzed with one‐way ANOVA followed by *LSD* or *Tamhani T2* post hoc test. *p < 0.05, **p < 0.01.

**Data S1.** Xxxxxx.

## Data Availability

Data will be made available on request.
