# Supplementary material for: 5‐HT7R Deficiency Alleviates ADP‐Heptose‐Induced Cognitive Impairment via Inhibiting Ferroptosis and Neuroinflammation in Mice
Source: CNS Neurosci Ther. 2025 Jun 12;31(6):e70455. doi: 10.1111/cns.70455 (PMC12159329; doi:10.1111/cns.70455)
Supplement: Supplementary file 1 — Figure S1. Effects of ADP‐hep on the protein expression of inflammatory factors in mice brain following ADP‐hep single intracerebroventricular administration. Protein levels of the TNF‐α, IL‐6 in the brain detected by ELISA. Data are expressed as mean ± SEM (n = 8/group). Data were analyzed with one‐way ANOVA followed by LSD or Tamhani T2 post hoc test. *p < 0.05, **p < 0.01 vs. Vehicle. Figure S2. Sorting and identification of microglia. (A, B) Schematic diagram of flow cytometry sorting of microglia from WT mice brain. (C) Immunofluorescence images show the expression of Iba‐1 for the identification of isolated cultured primary microglia from WT mice. Figure S3. GO/KEGG enrichment analysis and gene set enrichment analysis in the microglia sorted from the mice brain. (A–C) GO enrichment analysis. (D, E) KEGG enrichment analysis. (F–H) Gene set enrichment analysis. Figure S4. Genotyping of wild‐type (WT), 5‐HT7R heterozygous (5‐HT7R+/−), 5‐HT7R knockout (5‐HT7R−/−) mice. (A) Schematic diagram of mouse 5‐HT7R agarose gel electrophoresis results and (B) agarose gel electrophoresis results of part of mouse genotypes. Figure S5. Representative immunofluorescence images and quantitative analysis of 5‐HT7R and NeuN in the cerebral cortex or hippocampal CA1 subregions in the mice following 7 consecutive days of ADP‐hep intracerebroventricular administration (A–C). Scale bar = 50 μm. The data were expressed as mean ± SEM (n = 4/group). Data were analyzed with two‐tailed Student’s t‐test. **p < 0.01 vs. Vehicle group. Figure S6. Representative immunofluorescence images and quantitative analysis of 5‐HT7R and Iba1 in the cerebral cortex or hippocampal CA1 subregions in the mice following 7 consecutive days of ADP‐hep intracerebroventricular administration (A–C). Scale bar = 50 μm. The data were expressed as mean ± SEM. (n = 4/group). Data were analyzed with two‐tailed Student’s t‐test. **p < 0.01 vs. Vehicle group. Figure S7. Quantitative analyses of co‐localization of Ferrti [file CNS-31-e70455-s001.zip › cns70455-sup-0001-Figures.docx]

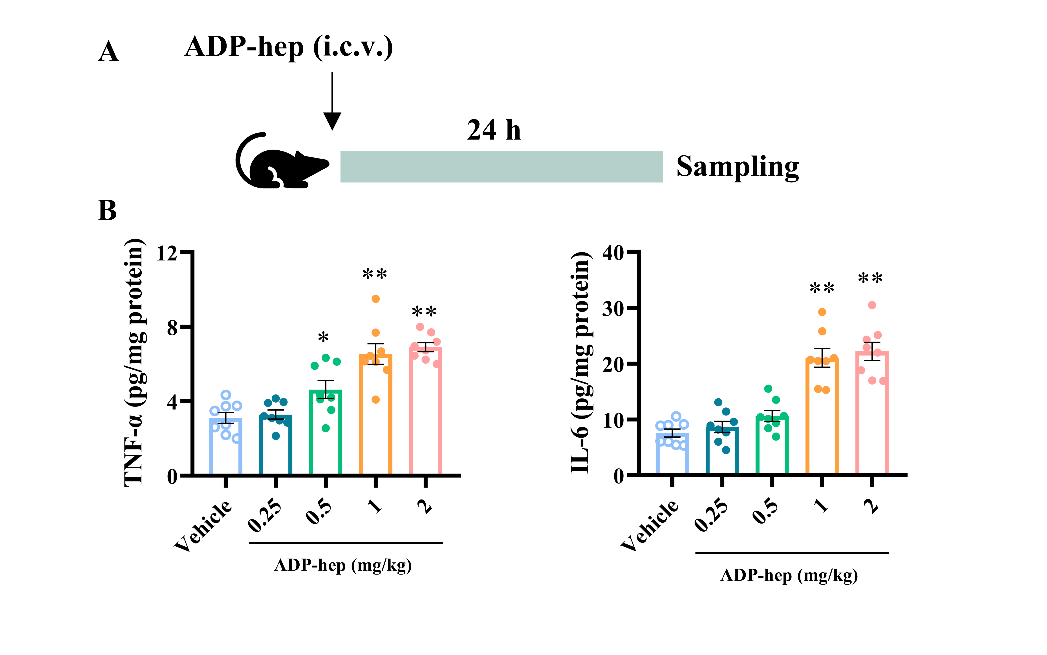


**Fig. S1.** Effects of ADP-hep on the protein expression of inflammatory factors in mice brain following ADP-hep single intracerebroventricular administration. Protein levels of the TNF-α, IL-6 in the brain detected by ELISA. Data are expressed as mean ± SEM (n = 8/group). Data were analyzed with one-way ANOVA followed by *LSD* or *Tamhani T2* post hoc test. ^*^*P* < 0.05, ^**^*P* < 0.01 vs. Vehicle.


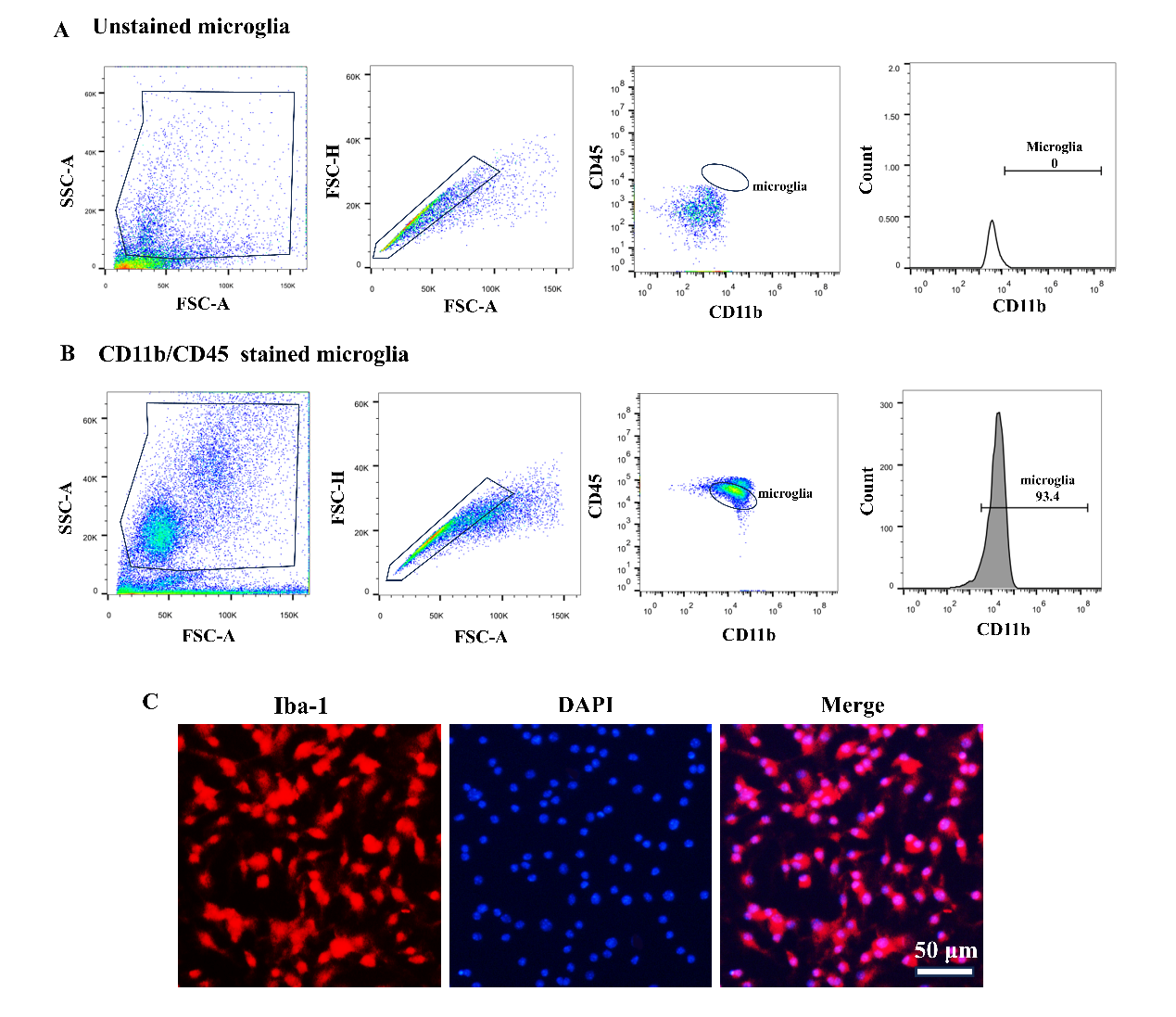


**Fig. S2.** Sorting and identification of microglia. (A-B) Schematic diagram of flow cytometry sorting of microglia from WT mice brain. (C) Immunofluorescence images show the expression of Iba-1 for the identification of isolated cultured primary microglia from WT mice.


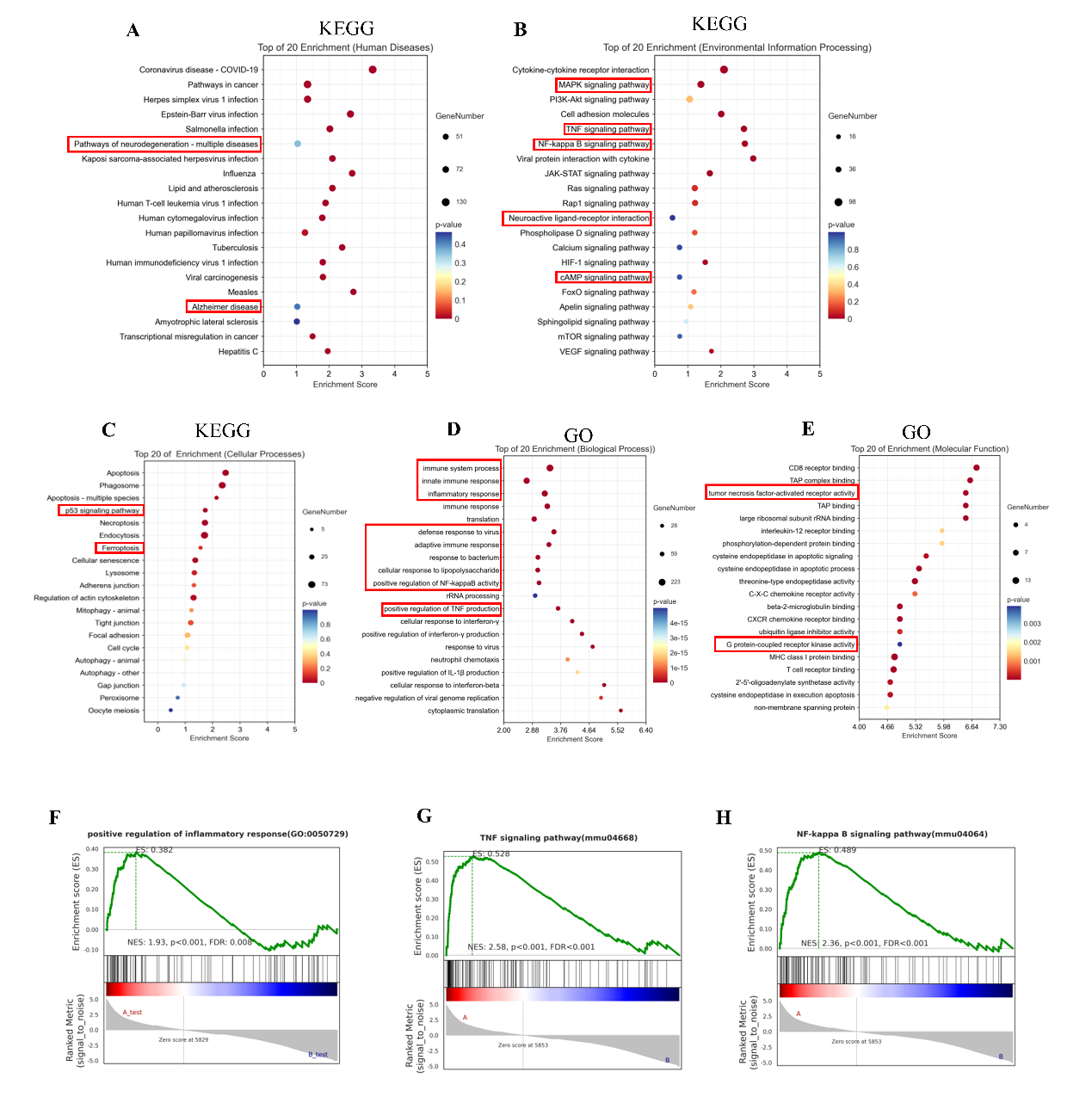


**Fig. S3.** GO/KEGG enrichment analysis and gene set enrichment analysis in the microglia sorted from the mice brain. (A-C) GO enrichment analysis. (D-E) KEGG enrichment analysis. (F-H) Gene set enrichment analysis.


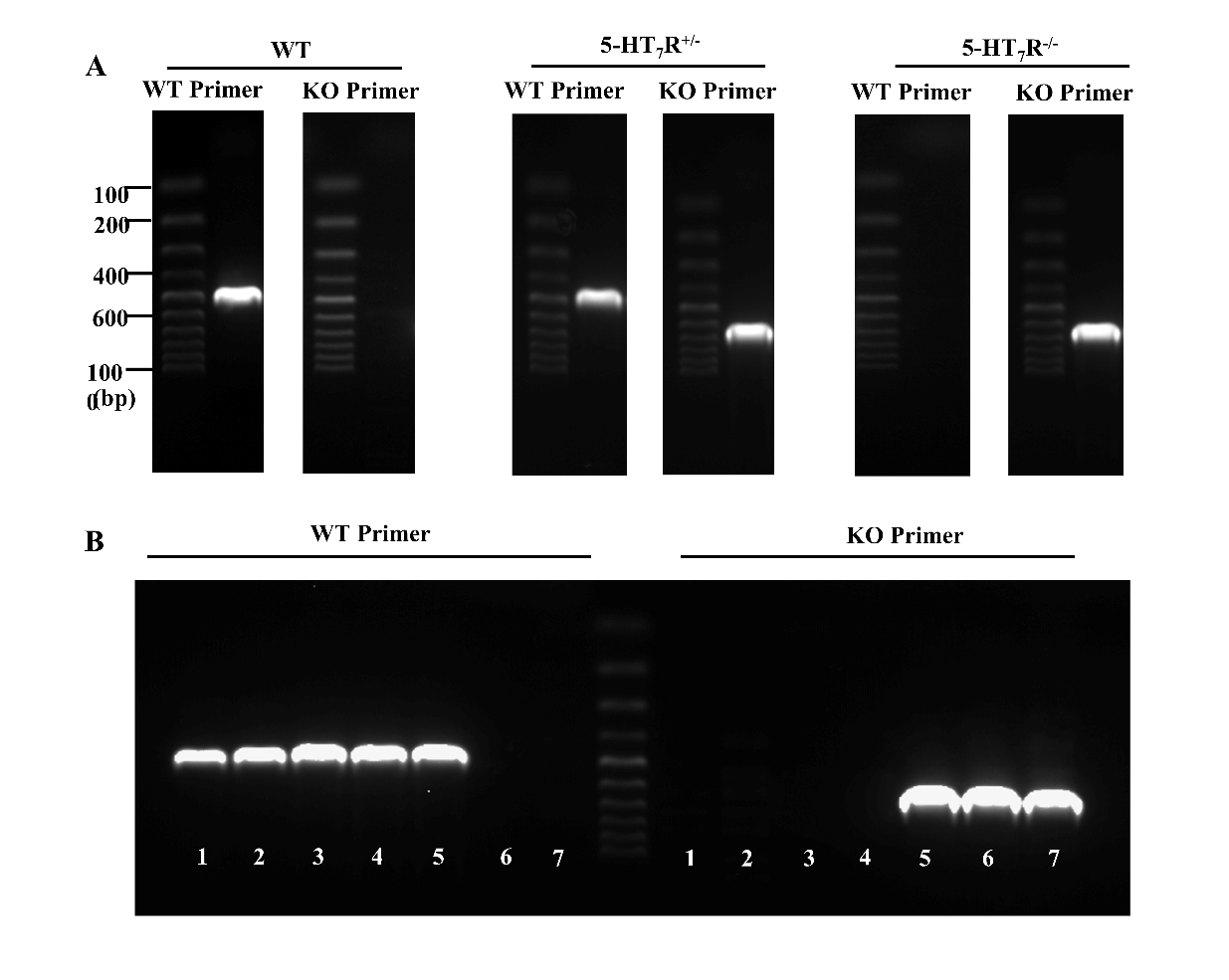


**Fig. S4.** Genotyping of wild-type (WT), 5-HT_7_R heterozygous (5-HT_7_R^+/-^), 5-HT_7_R knockout (5-HT_7_R^-/-^) mice. (A) Schematic diagram of mouse 5-HT_7_R agarose gel electrophoresis results; (B) Agarose gel electrophoresis results of part of mouse genotypes.


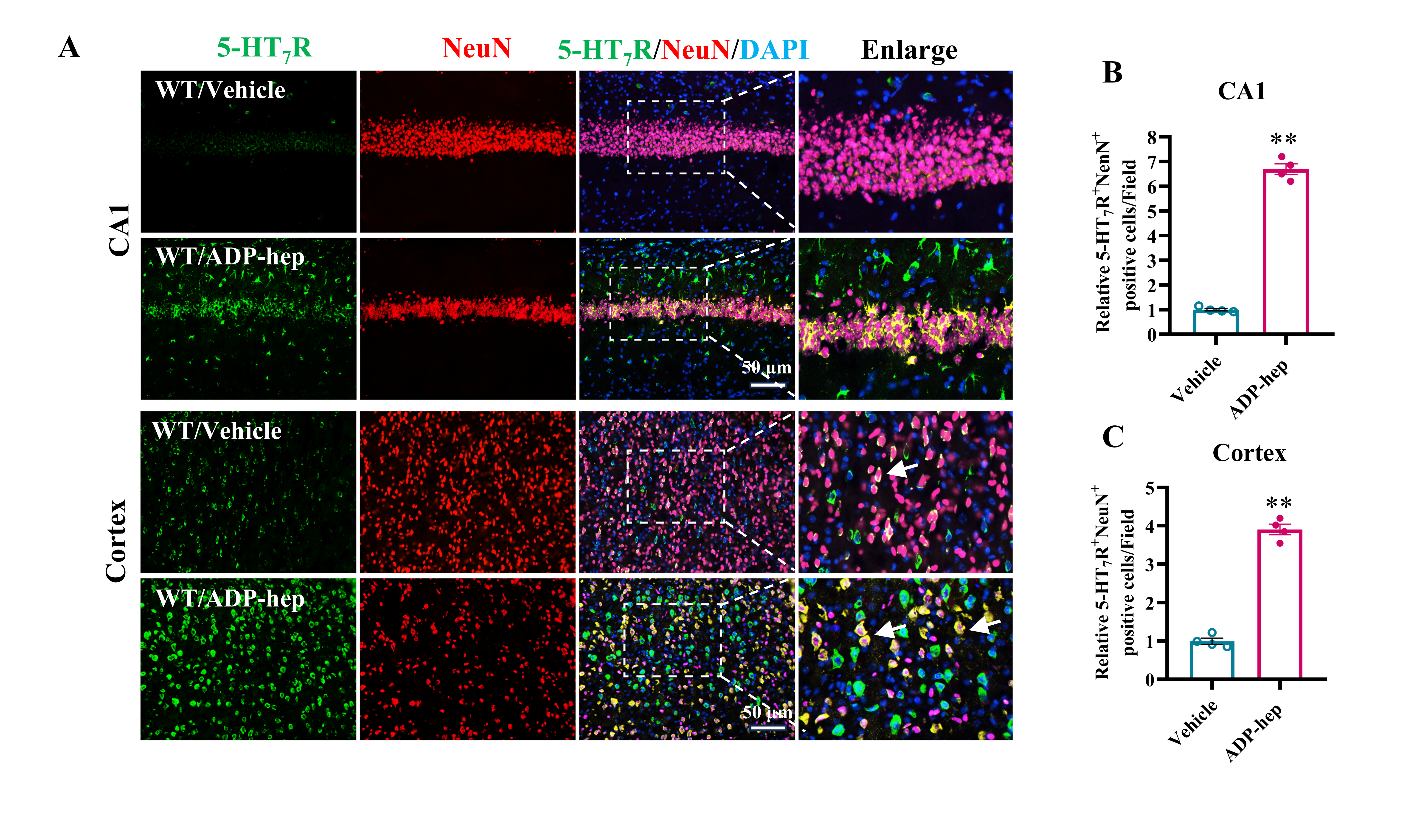


**Fig S5**. Representative immunofluorescence images and quantitative analysis of 5-HT_7_R and NeuN in the cerebral cortex or hippocampal CA1 subregions in the mice following 7 consecutive days of ADP-hep intracerebroventricular administration (A-C). Scale bar = 50 μm. The data were expressed as mean ± SEM. (n=4/group). Data were analyzed with two-tailed Student's *t*-test. ^**^*P* < 0.01 vs. Vehicle group.


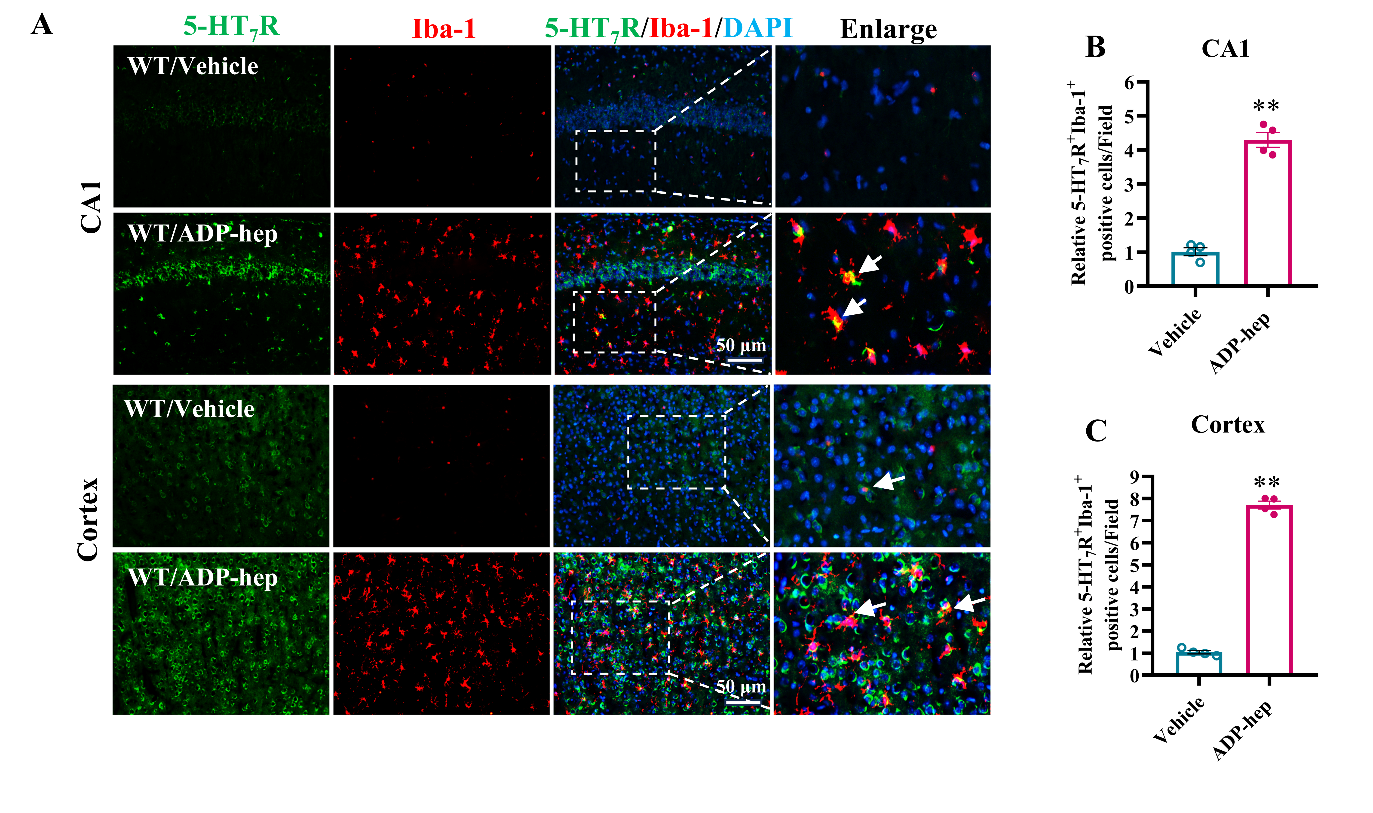


**Fig S6.** Representative immunofluorescence images and quantitative analysis of 5-HT_7_R and Iba1 in the cerebral cortex or hippocampal CA1 subregions in the mice following 7 consecutive days of ADP-hep intracerebroventricular administration (A-C). Scale bar = 50 μm. The data were expressed as mean ± SEM. (n=4/group). Data were analyzed with two-tailed Student's *t*-test. ^**^*P* < 0.01 vs. Vehicle group.


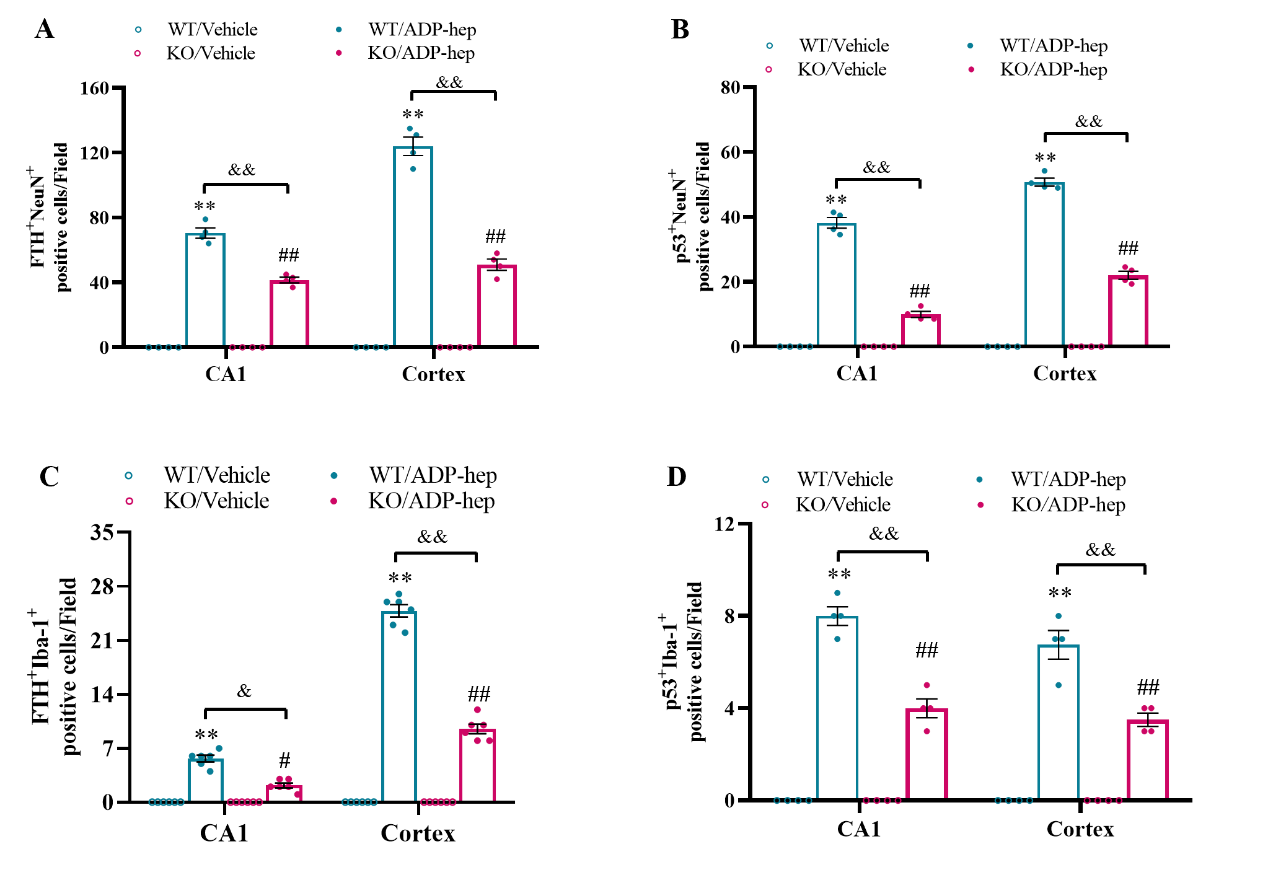


**Fig S7**. Quantitative analyses of co-localization of Ferrtin and NeuN (A), p53 and NeuN (B), Ferrtin and Iba-1 (C), p53 and Iba-1 (D) in the cerebral cortex or hippocampal CA1 subregions. Data were expressed as mean ± SEM. (n=4/group). Data were analyzed with two-way ANOVA followed by the *Bonferroni-Holm* post hoc test. ^**^*P* < 0.01 vs. WT/vehicle group, ^##^*P* < 0.01 vs. KO/ADP-hep, ^&^*P* < 0.05, ^&&^*P* < 0.01 vs. KO/ADP-hep.


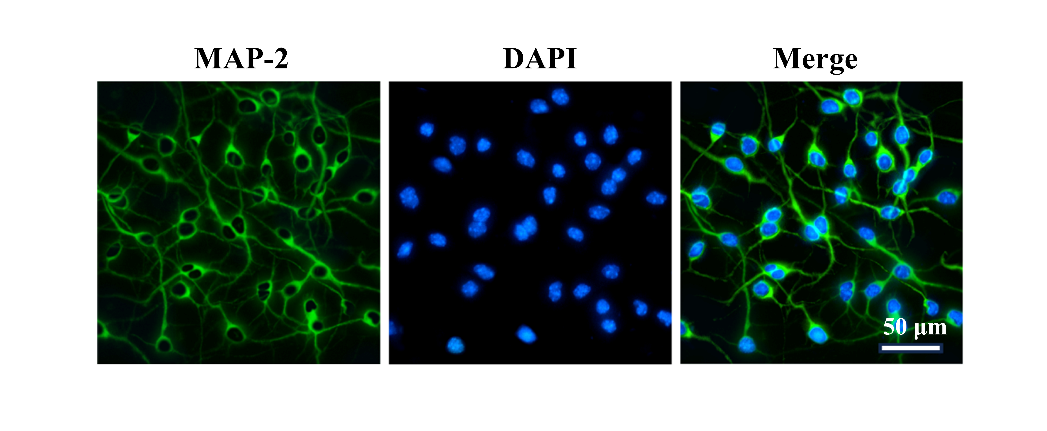


**Fig S8**. Identification of primary neuronal cells. Immunofluorescence images show the expression of MAP-2 for the identification of isolated cultured primary neuronal cells from WT mice. Scale bar = 50 μm.

**
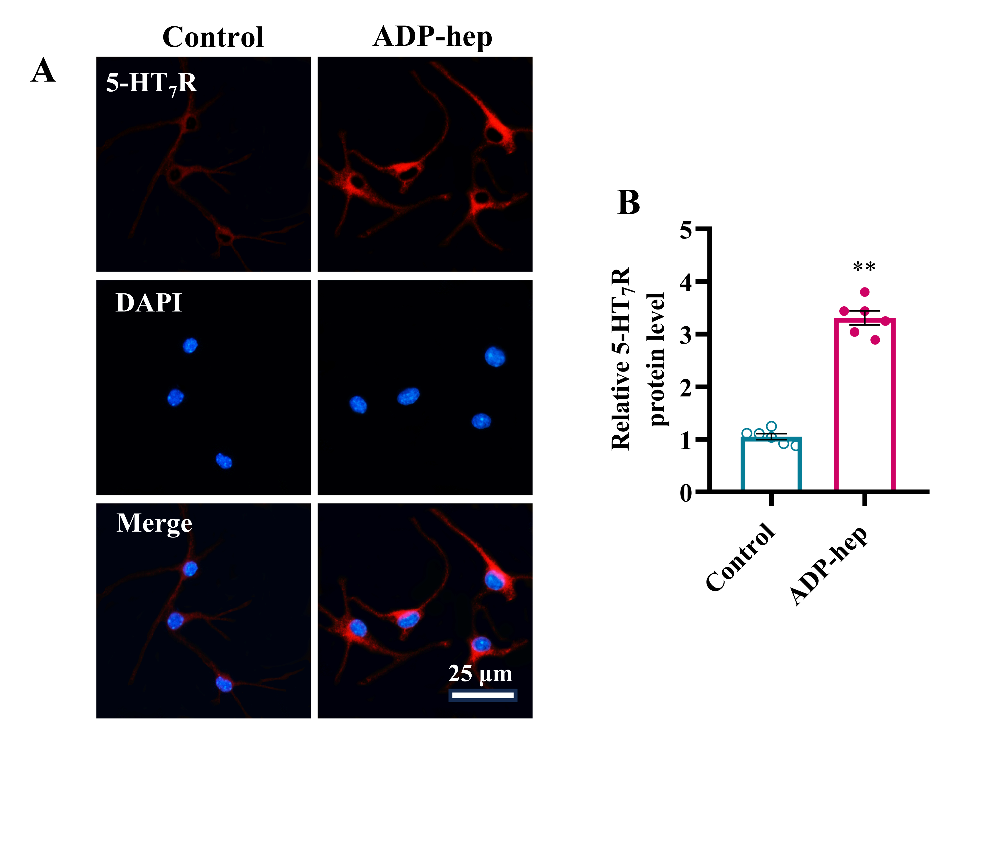
Fig S9**. The relative expression of 5-HT_7_R in primary neuronal cells were detected by immunofluorescence after ADP hep (100 μM) treatment for 24 hours. The data were expressed as mean ± SEM, *n* = 6/group. Data were analyzed with two-tailed Student's *t*-test. ^**^*P* < 0.01 vs. Control group.


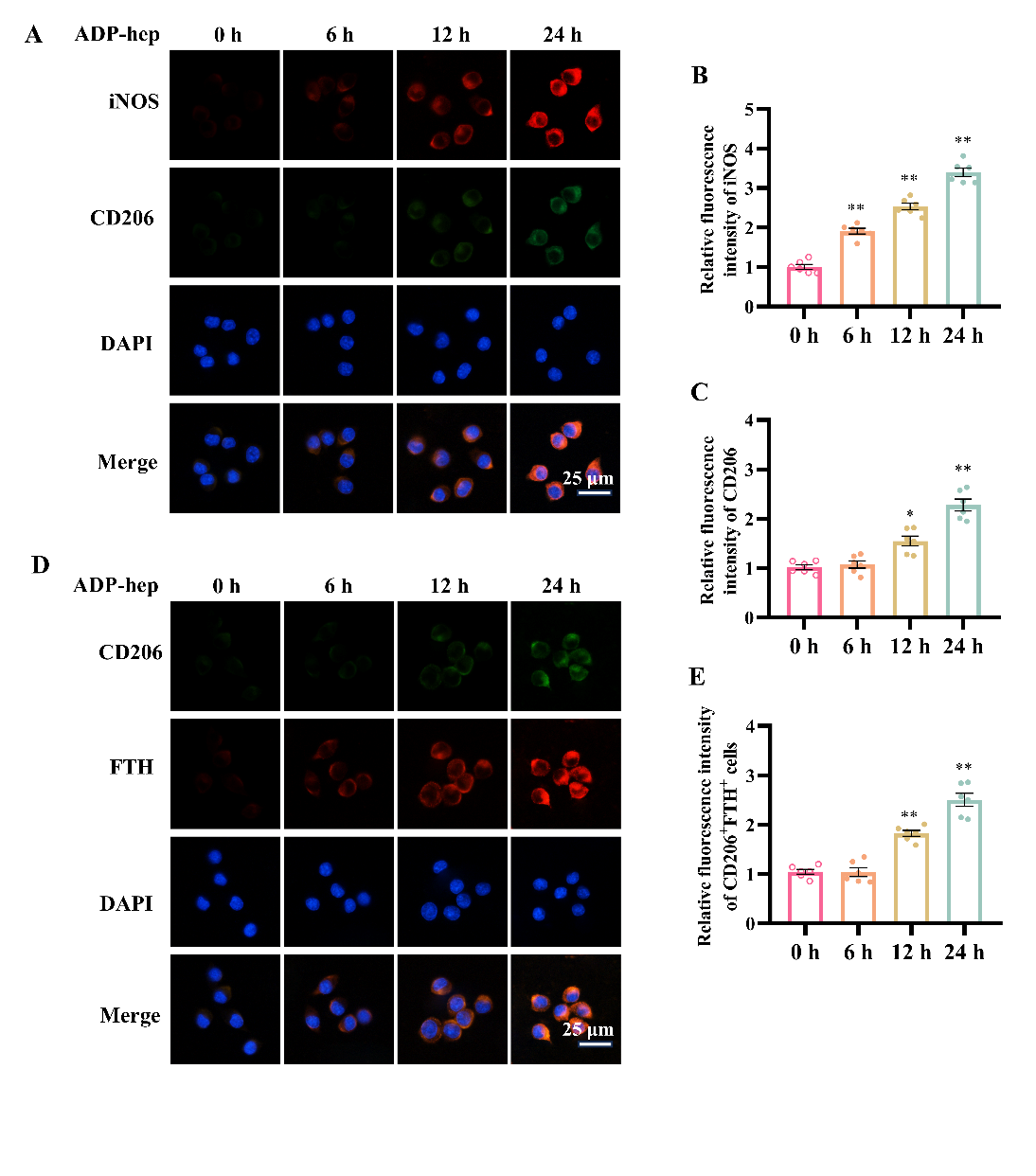


**Fig S10**. Temporal dynamics of M1/M2 microglial phenotypic polarization. BV2 cells were incubated with ADP-hep (100 μM) for 0h, 6h, 12h and 24h. (A-C) Representative immunofluorescence images and quantitative analysis of iNOS and CD206 (400×), CD206 (green), iNOS (red). (D-E) Representative immunofluorescence images and quantitative analysis of CD206 and FTH (400×), CD206 (green), FTH (red). The data were expressed as mean ± SEM, *n* = 6/group. Data were analyzed with two-tailed Student's *t*-test. **P* < 0.05, ***P* < 0.01 vs. 0 h group.


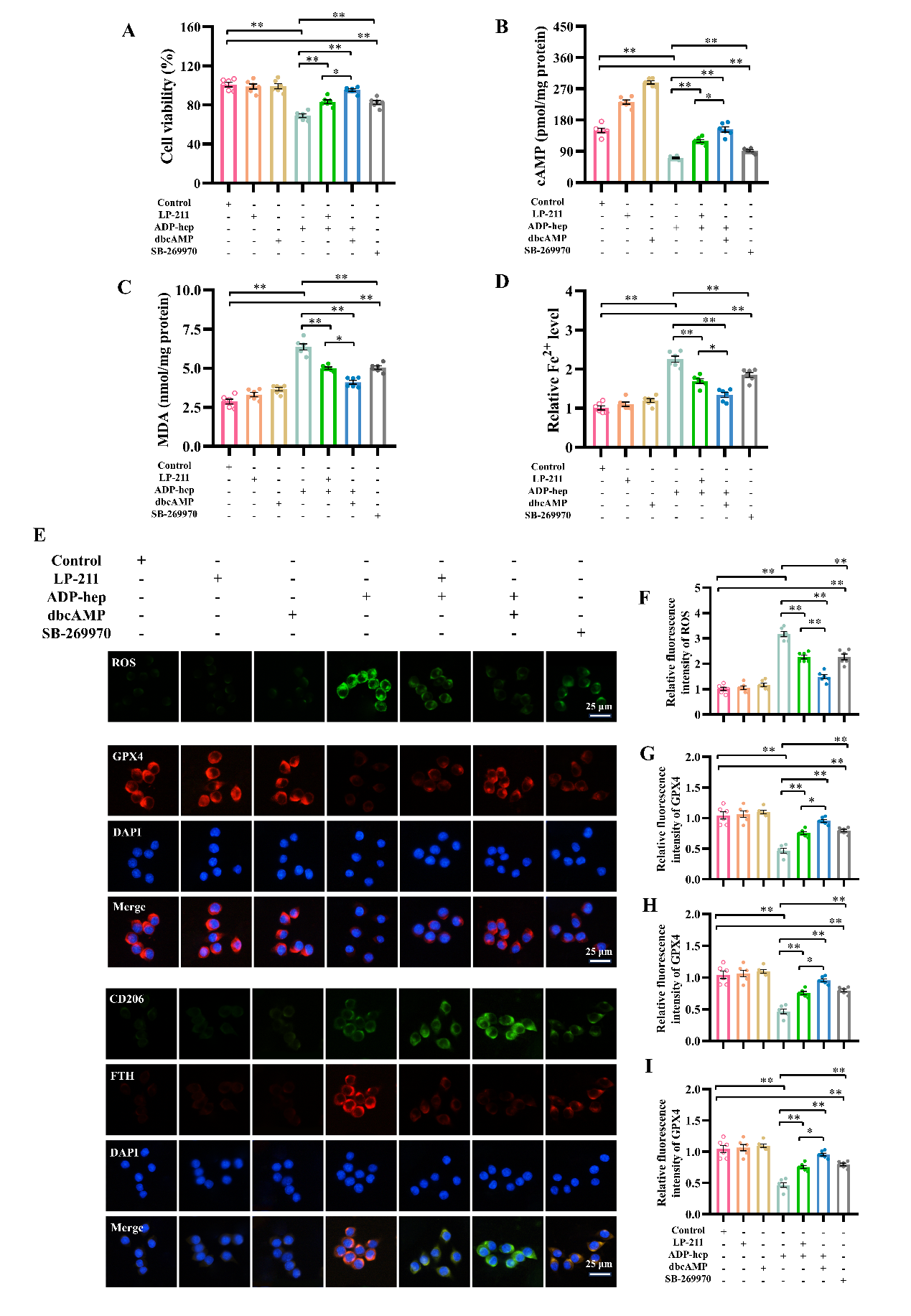


**Fig S11**. Effects of 5-HT_7_R agonist, dibutyryl cAMP, 5-HT_7_R antagonist on ADP-hep induced ferroptosis in BV2 cells. (A) The survival rate of BV2 cells. (B) The cAMP content, (C) the content of MDA and (D) the level of Fe^2+^ in BV2 cells. (E-I) Representative immunofluorescence images and quantitative analysis of ROS, GPX4, CD206, FTH (400 ×), ROS (green), GPX4 (red), CD206 (green), FTH (red), DAPI (blue). The data were expressed as mean ± SEM, *n* = 6/group. Data were analyzed with one-way ANOVA followed by *LSD* or *Tamhani T2* post hoc test. **P* < 0.05, ***P* < 0.01.
