# Supplementary material for: 5‐HT7R Deficiency Alleviates ADP‐Heptose‐Induced Cognitive Impairment via Inhibiting Ferroptosis and Neuroinflammation in Mice
Source: CNS Neurosci Ther. 2025 Jun 12;31(6):e70455. doi: 10.1111/cns.70455 (PMC12159329; doi:10.1111/cns.70455)

Full unedited gel/blot for Fig.1 K

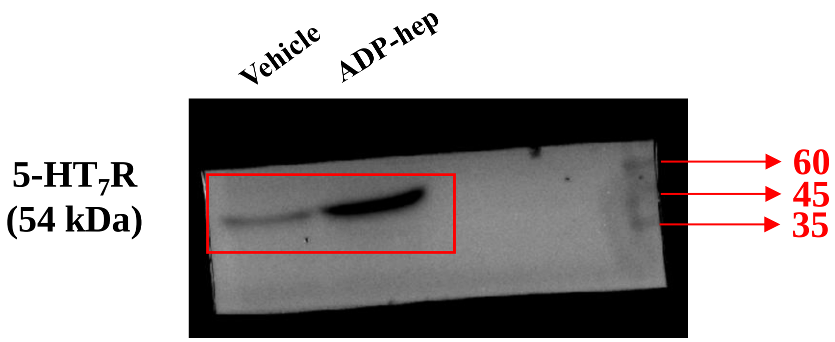

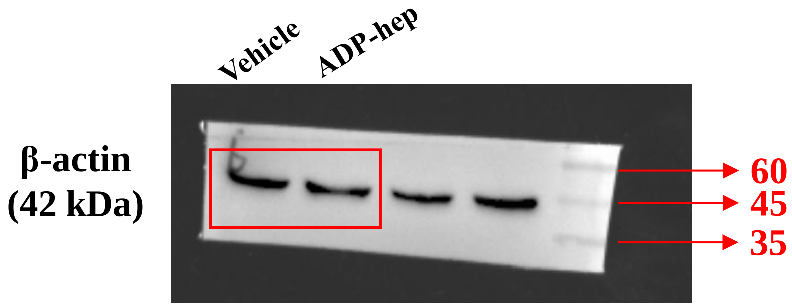

Full unedited gel/blot for Fig.2 B

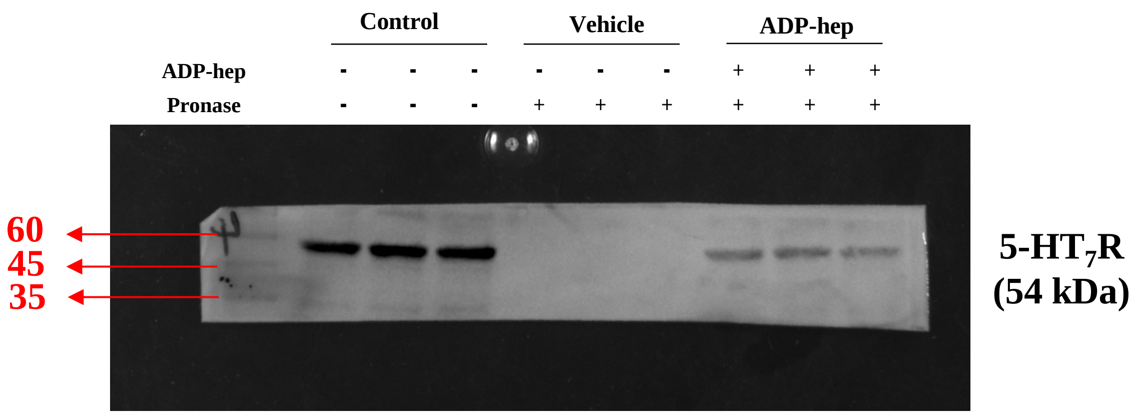

Full unedited gel/blot for Fig.2 C

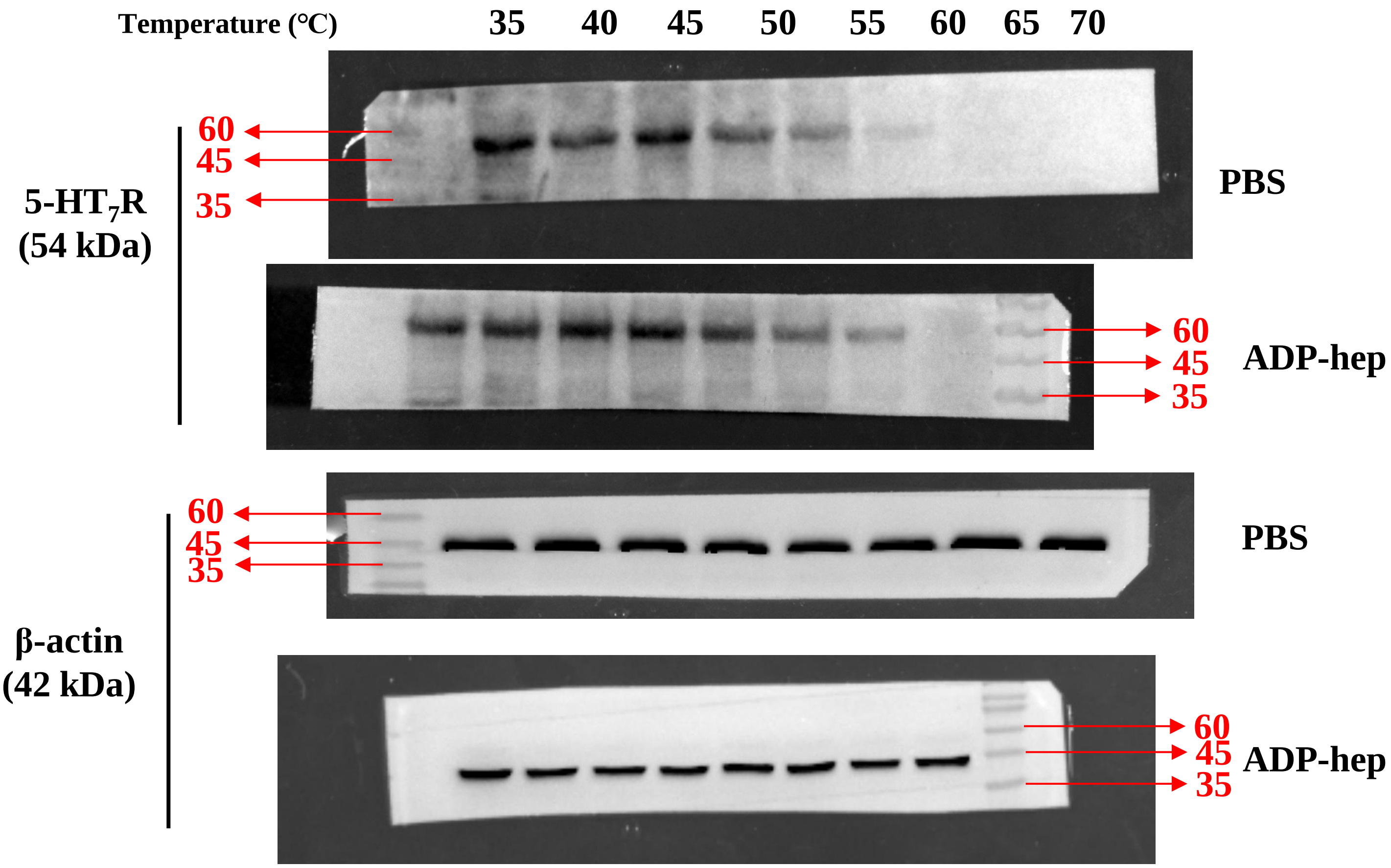

Full unedited gel/blot for Fig.3 J

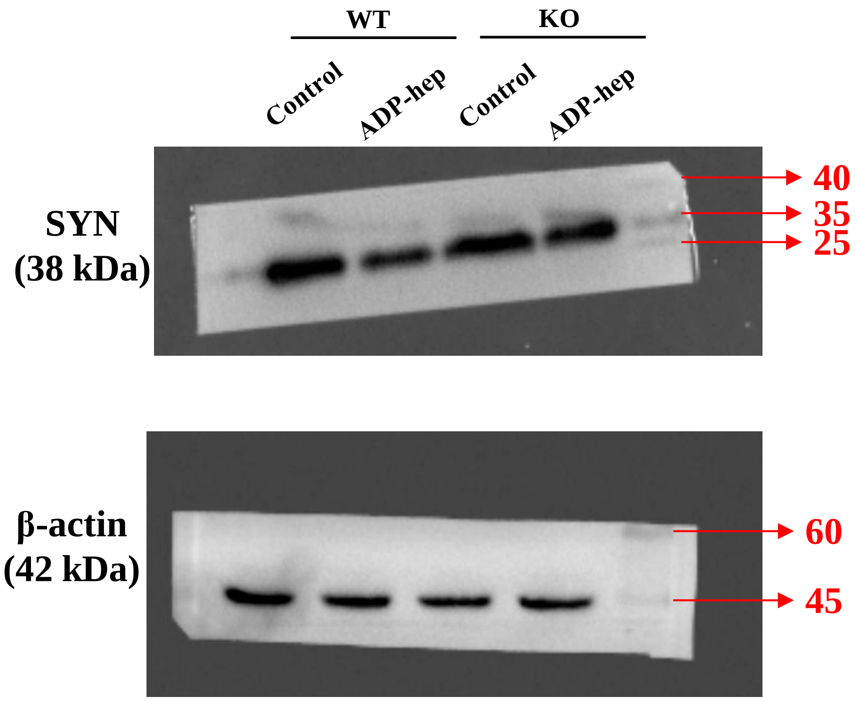

Full unedited gel/blot for Fig.4 B

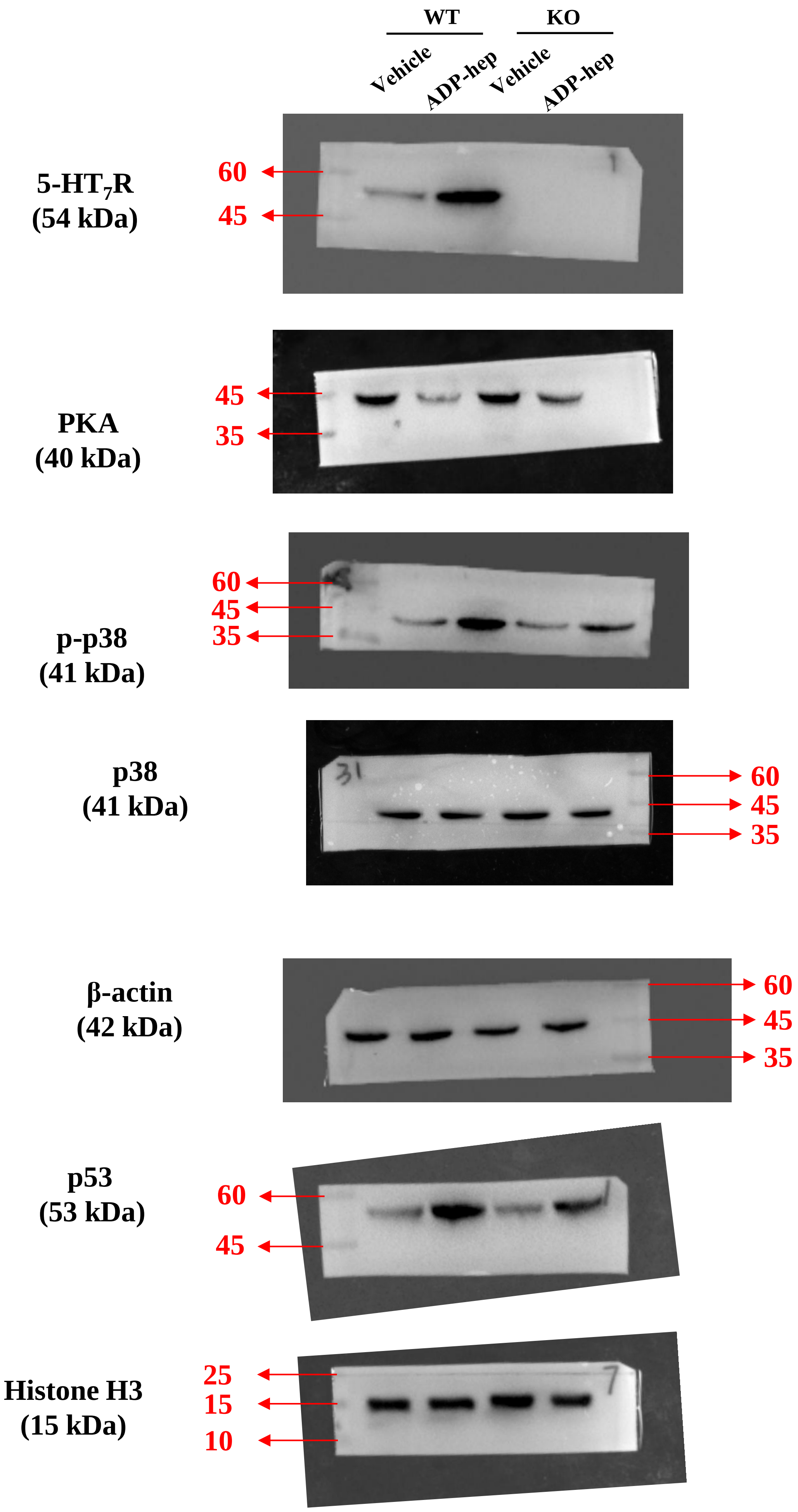

Full unedited gel/blot for Fig.4 B

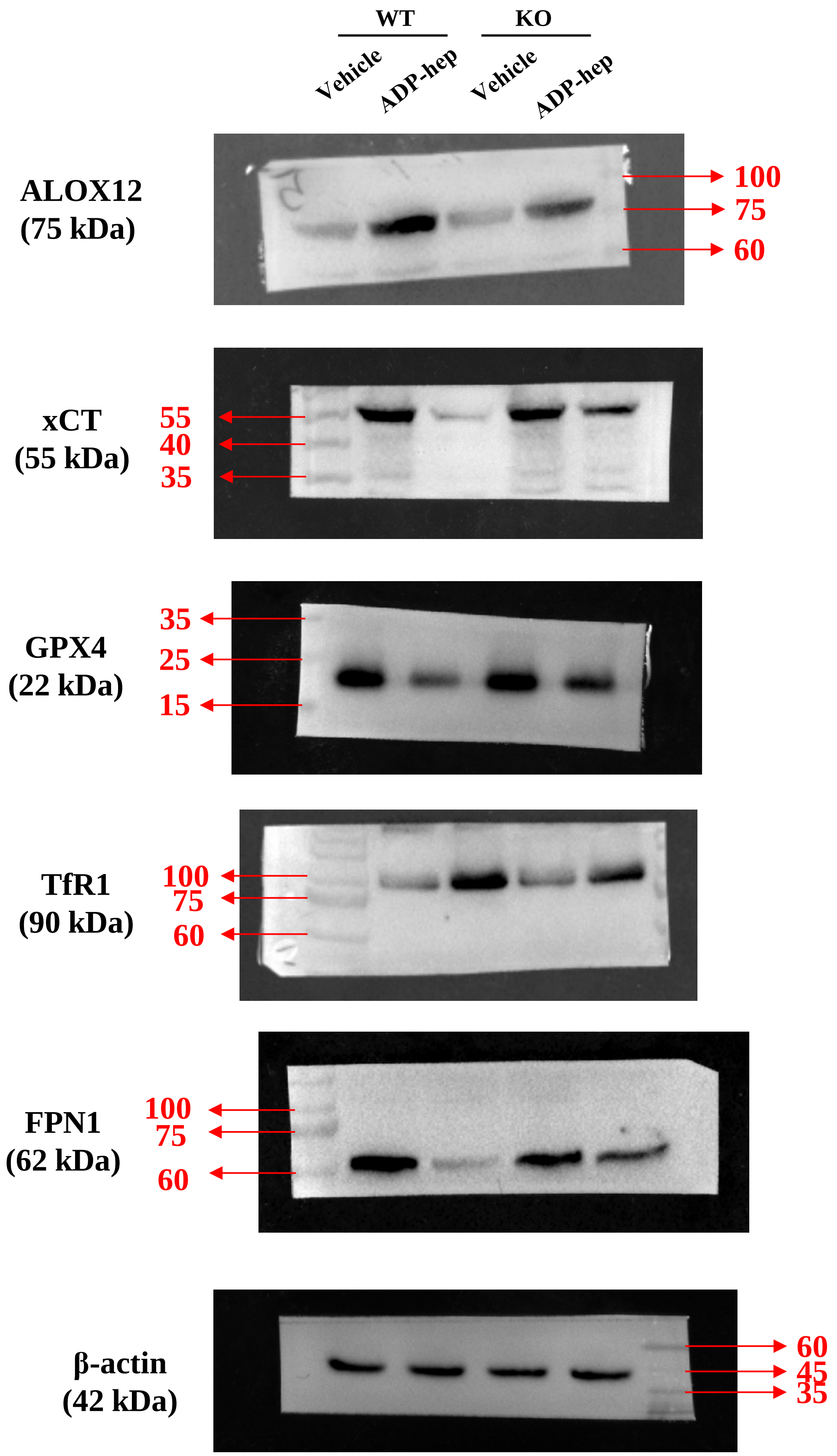

Full unedited gel/blot for Fig.5 L

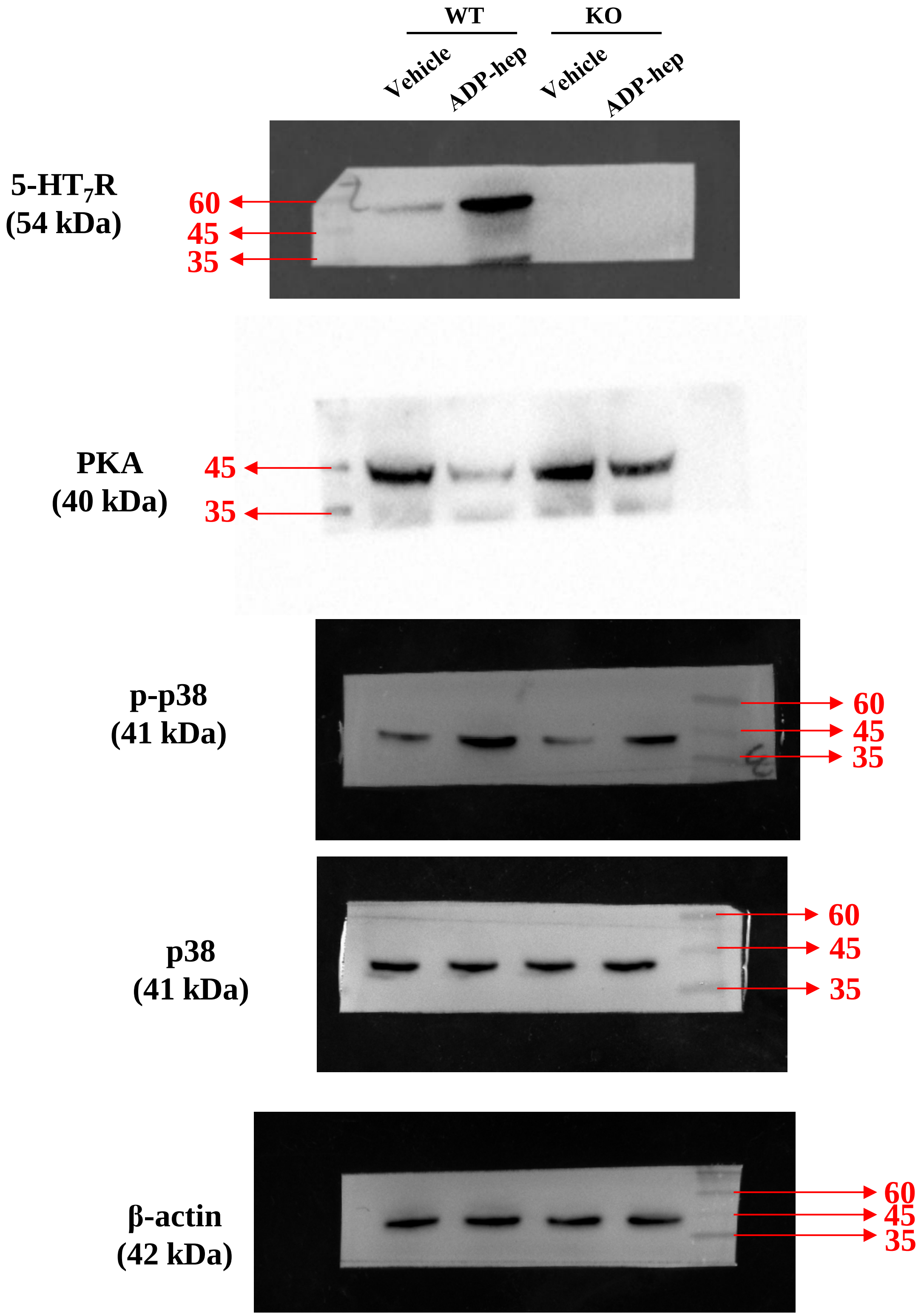

Full unedited gel/blot for Fig.5 L

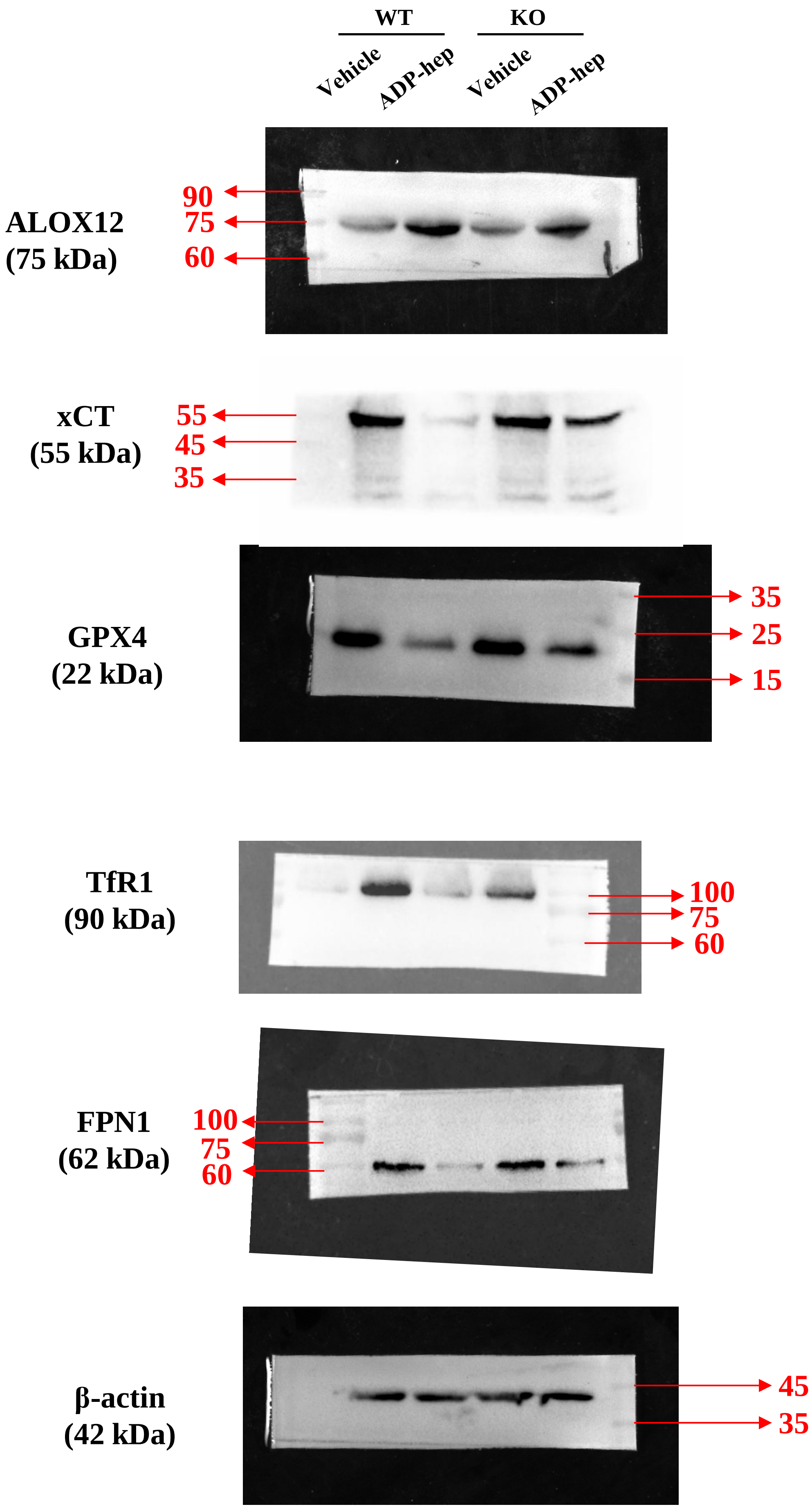

Full unedited gel/blot for Fig.6 L

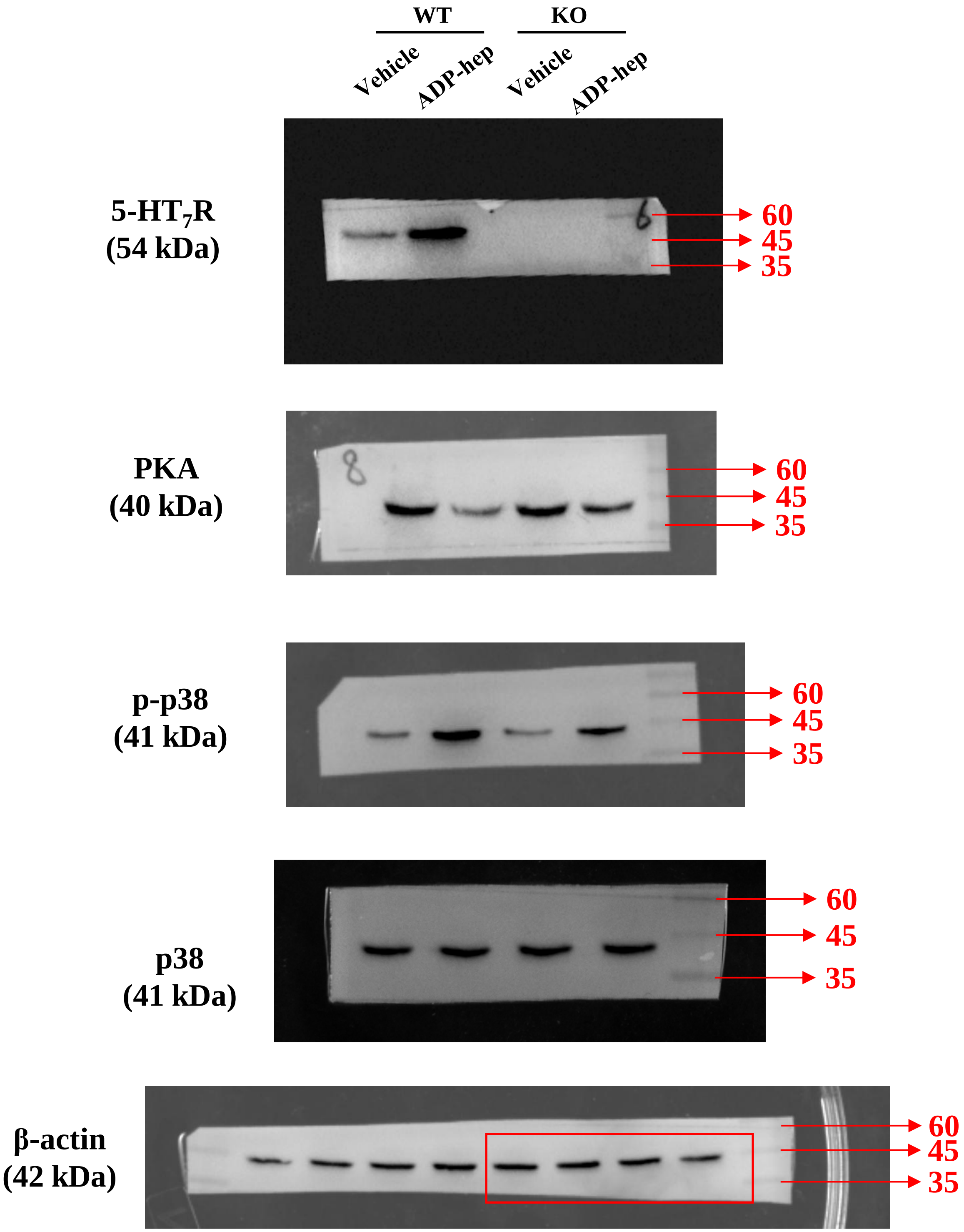

Full unedited gel/blot for Fig.6 L

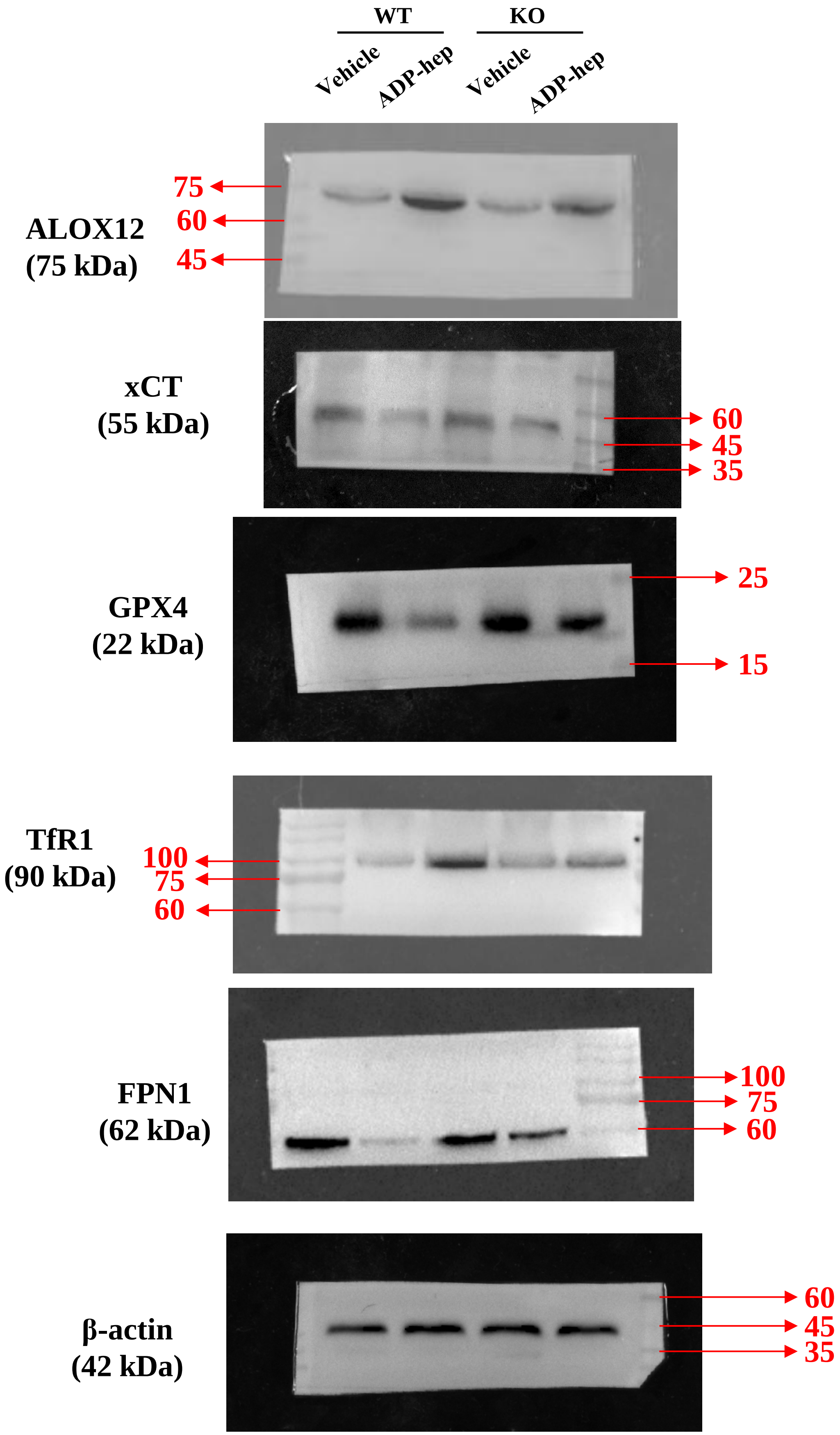

Full unedited gel/blot for Fig.8 A

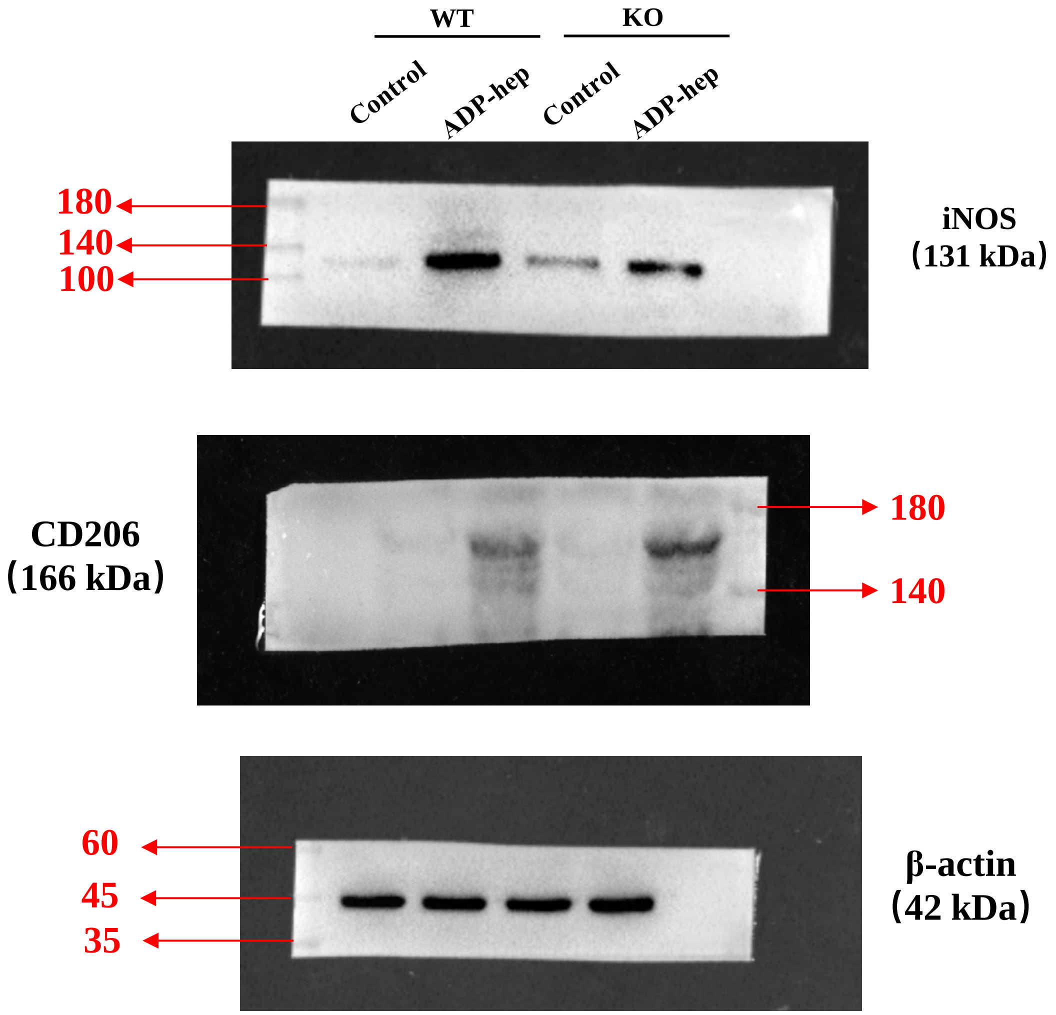

Supplement: Supplementary file 1 — Figure S1. Effects of ADP‐hep on the protein expression of inflammatory factors in mice brain following ADP‐hep single intracerebroventricular administration. Protein levels of the TNF‐α, IL‐6 in the brain detected by ELISA. Data are expressed as mean ± SEM (n = 8/group). Data were analyzed with one‐way ANOVA followed by LSD or Tamhani T2 post hoc test. *p < 0.05, **p < 0.01 vs. Vehicle. Figure S2. Sorting and identification of microglia. (A, B) Schematic diagram of flow cytometry sorting of microglia from WT mice brain. (C) Immunofluorescence images show the expression of Iba‐1 for the identification of isolated cultured primary microglia from WT mice. Figure S3. GO/KEGG enrichment analysis and gene set enrichment analysis in the microglia sorted from the mice brain. (A–C) GO enrichment analysis. (D, E) KEGG enrichment analysis. (F–H) Gene set enrichment analysis. Figure S4. Genotyping of wild‐type (WT), 5‐HT7R heterozygous (5‐HT7R+/−), 5‐HT7R knockout (5‐HT7R−/−) mice. (A) Schematic diagram of mouse 5‐HT7R agarose gel electrophoresis results and (B) agarose gel electrophoresis results of part of mouse genotypes. Figure S5. Representative immunofluorescence images and quantitative analysis of 5‐HT7R and NeuN in the cerebral cortex or hippocampal CA1 subregions in the mice following 7 consecutive days of ADP‐hep intracerebroventricular administration (A–C). Scale bar = 50 μm. The data were expressed as mean ± SEM (n = 4/group). Data were analyzed with two‐tailed Student’s t‐test. **p < 0.01 vs. Vehicle group. Figure S6. Representative immunofluorescence images and quantitative analysis of 5‐HT7R and Iba1 in the cerebral cortex or hippocampal CA1 subregions in the mice following 7 consecutive days of ADP‐hep intracerebroventricular administration (A–C). Scale bar = 50 μm. The data were expressed as mean ± SEM. (n = 4/group). Data were analyzed with two‐tailed Student’s t‐test. **p < 0.01 vs. Vehicle group. Figure S7. Quantitative analyses of co‐localization of Ferrti [file CNS-31-e70455-s001.zip › cns70455-sup-0003-DataS2.pdf]
